# Perylene bisbenzimidazole nonlinear dielectric material for energy storage†

**DOI:** 10.1039/c8ra08873j

**Published:** 2019-01-02

**Authors:** Samuel J. Hein, Carine Edder, Marta Kowalczyk, Andrey Borzenko, Lev Mourokh, Pavel Lazarev

**Affiliations:** Capacitor Sciences Inc. 1530 O'Brien Drive, Suite B Menlo Park CA 94025 USA; Natural Sciences Department, LaGuardia Community College, City University of New York 31-10 Thomson Avenue Long Island City NY 11101 USA; Department of Physics, Queens College of the City University of New York Flushing NY 11367 USA

## Abstract

A novel perylene bisbenzimidazole comprising both donor and acceptor functional groups was designed, synthesized, and characterized. This structure exhibits potentially useful physical properties, including a nonlinear dielectric response to an increasing electric field. This material can be used in energy storage devices as the dielectric part of a capacitor. Energy storage devices based on film capacitors are targeting applications in a wide range of industrial, residential and transportation systems.

## Abstract 2

We synthesized and characterized an organic molecule which can serve as a unit for high-density energy storage.

## Graphical abstract

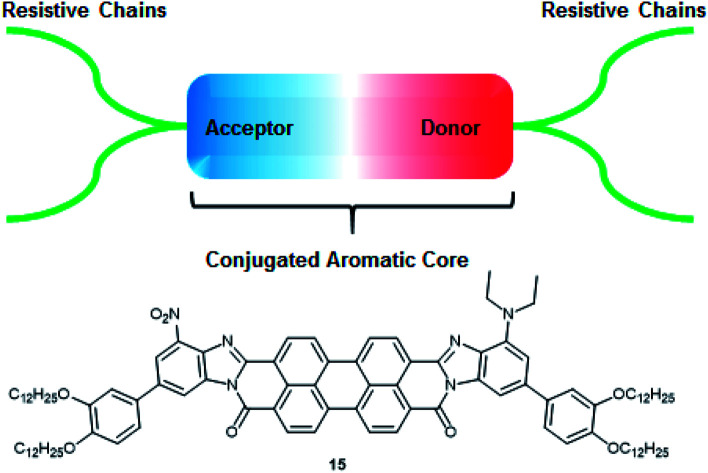

Capacitors are among the simplest devices that store electrical energy and feature high charge–discharge rates, high power density, and a wide range of operating temperatures.^1^ As a result, they have found use in many electronic devices and in numerous industrial applications. However, while capacitors feature high power density, they do not compete with the high gravimetric energy density of batteries, precluding their use as long-term energy storage devices. This disadvantage originates from the low polarizability of conventional dielectric materials found in modern industrial capacitors. We are developing new dielectric materials with high polarizability, high resistivity, and high breakdown voltage. These materials will widen the scope of capacitor applications to a variety of energy storage devices including transportation, industry, and residential applications.

For traditional materials, dielectric polarizability is a constant value with stored energy sharing a linear relationship to the dielectric permittivity of the material. At high electric fields, however, many materials exhibit a nonlinear dielectric constant.^2^ Commonly, these materials consist of highly polarizable molecules with electron-donating and/or electron-withdrawing groups on opposite sides of a large conjugated core. These groups can increase polarizability and further impart directionality to the delocalized electrons when an electric field is applied.

Here we present the general structure for a non-linear dielectric chromophore, that we named dielectrophore, that would bring the combination of required properties into capacitors: high permittivity, resistivity, and breakdown strength, as well as good processability and mechanical flexibility.^3^ The main three components that build these properties are: the polyaromatic core, allowing π–π stacking within the material as well as high polarizability, donor and acceptor groups at each end of the conjugated core increasing the first hyperpolarizability, and insulating subunits that prevent current leakage within the capacitor (Fig. 1). In such structures, the cores are predominantly planar polycyclic molecular system which forms column-like supramolecular stacks by π–π interactions.

**Fig. 1:**
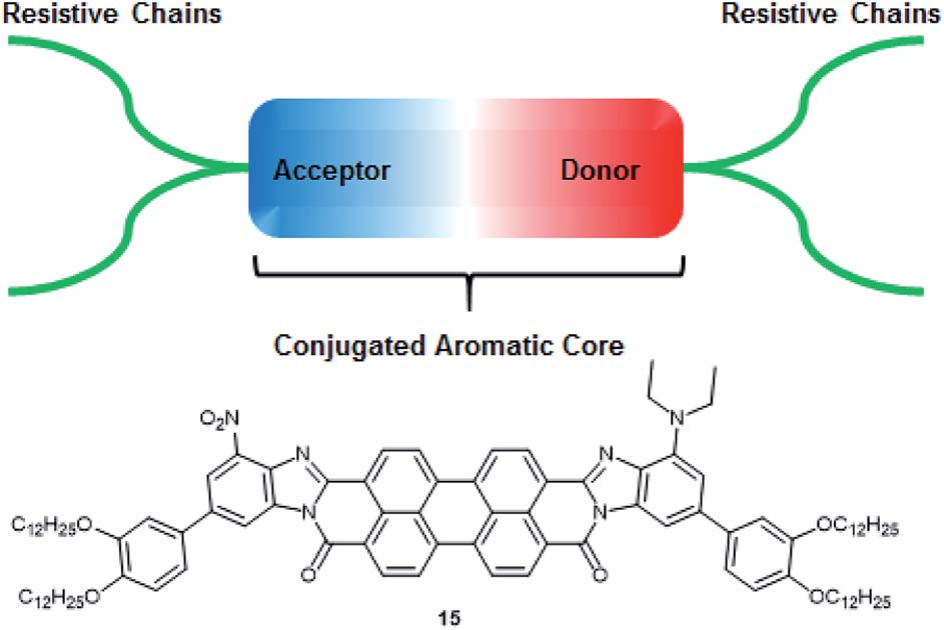
General representation of an ideal dielectrophore, such as 15.

Perylene-3,4,9,10-tetracarboxylic acid diimides (PDIs) are among the most extensively used cores for studies of π–π stacking and columnar liquid crystalline structures. It is noteworthy that PDI-based molecules have been widely used as colorants and dyes, as well as chromophores for optoelectronic applications such as photovoltaics and organic field effect transistors. This is due to the combination of their optoelectronic properties, such as large extinction coefficients, high fluorescent quantum yields, strong electron-accepting ability (N-type semiconductors), and high thermal and chemical stability.^4^ Most of these properties originate from the delocalization of the π electrons of the PDI's. In addition, the modular structure and straightforward synthesis of PDI-based molecules streamline the modification of their structures, which helps to bring all the required properties of our dielectrophores.

Our theoretical studies demonstrated that linear polarizability of the PDIs can be strongly increased by transforming the diimide functions into the more conjugated benzimidazole derivatives and adding donor (NEt_2_) and acceptor groups (NO_2_) at the opposite sides of a molecule.^5^ Considering the predicted design, we propose molecule 15 as our target molecule (Fig. 1). Long alkyl chains are added *via* phenyl linkers at both sides of the molecule and bring significant impact into the resistivity of the potential capacitor and help with processability during the synthetic manipulations and film coating.

One of the main advantages of 15 is the modularity of its synthesis, which can be performed as a step-wise addition of two substituted *o*-phenylenediamines to 13 (Scheme 1). 13 was synthesized according to a modified procedure reported by Xiao and co-workers (outlined in Scheme 1).^6^ In our synthesis, 3 is accessed through a 2-step derivatization of catechol (1) through an alkylation followed by a C–H activated borylation.^7^ The electron-acceptor diamine (7) was synthesized from 2,5-dinitroaniline (4) *via* bromination with NBS, followed by Suzuki coupling with 6, and further reduction of one of the nitro groups with ammonium sulfide. The electron donating diamine (12) was synthesized from 3-bromo-2,5-difluoronitrobenzene (8) *via* nucleophilic aromatic substitution at room temperature, followed by Suzuki coupling with 3 to isolate 10. A nucleophilic aromatic substitution with benzylamine at elevated temperature yields 11 and a deprotection/reduction using Pd(OH)_2_/C and H_2_ at 55 psi afforded 12. 14 was obtained in two steps by stirring diamine 7 with 13 in molten imidazole at 140 °C for 12 hours then further treating the product with an excess of *para*-toluenesulfonic acid in toluene at 100 °C to afford 14 in 37% overall yield (Scheme 1).

**Scheme 1:**
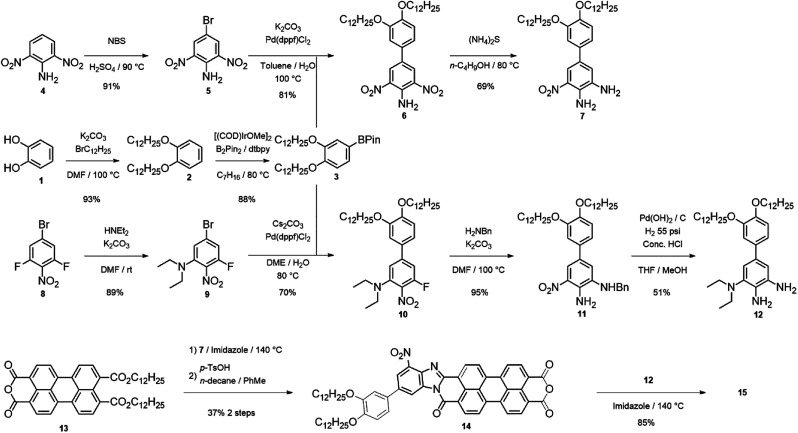
Synthesis of 15.

15 was made through the condensation of mono-anhydride 14 with diamine 12 with the presence of zinc acetate in quinoline at 140 °C for 12 hours. Upon precipitation into methanol, 15 was isolated as a dark purple solid. It is important to mention that reactions of 13 with 7 and then 14 with 12 are likely not regioselective, even though steric factors favour the formation of the isomers 15a and 15b (Scheme 2) with donor/acceptor groups located farther from carboxamide group, especially in the case of the bulkier diethylamino group. Indeed, six additional isomers may be present, in addition to 15a and 15b (15c–h, see ESI†). Column chromatography to separate these regioisomeric mixtures is shown to be quite challenging.^8^ Considering our theoretical studies, that demonstrated both *syn*- and *anti*-isomers to have similar predicted polarization values, separation of individual isomers is unnecessary.^5^

**Scheme 2:**
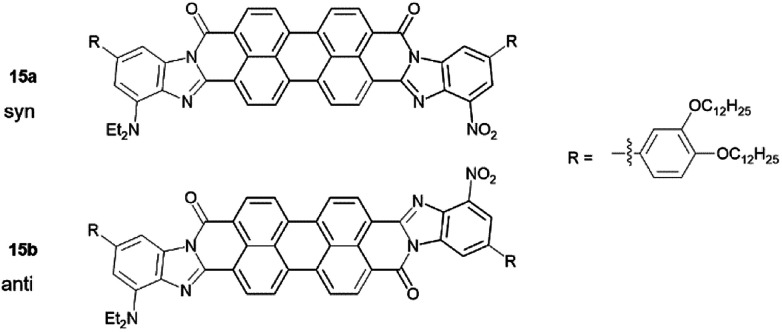
Main isomers of 15.

15 was characterized with UV-vis and FTIR spectroscopic techniques and molecular weight was confirmed through mass spectrometry (see ESI†). FTIR of 15 shows the absence of the anhydride carbonyl stretches of 14 at 1769 and 1732 cm^−1^ respectively and the emergence of benzimidazole stretches at 1691, 1592, and 1573 cm^−1^.^9^^1^H NMR of 15 in 100% CDCl_3_ initially exhibit very broad signals, but when 10% dTFA is added this aggregation is broken up (see ESI†). The formation of the benzimidazole extends the length of conjugation considerably, this transformation can be clearly observed in the UV-vis spectra where the *λ*_max_ shifts from 523 nm for 13 to 555 nm for 14 (ESI†). The *λ*_max_ shifts even further towards the near infrared region (752 nm) upon addition of the donor block (12) and making 15 (Fig. 2A). The drastic broadening of the band from 500 to 900 nm for 15 is caused by the extended conjugation, the formation of π–π-aggregates in the solution and the presence of a mixture of isomers,^8*a*^ as well as intramolecular charge transfer (ICT) in this push–pull system.^10^ The UV-vis spectrum in chloroform was predicted using a B3LYP/6-31H level of theory and compared with the experimental data (see ESI†). The computed spectrum identifies three major bands at 409, 552, and 850 nm, respectively, in some agreement with the observed *λ*_max_ at 395, 525, and 752 nm (ESI†). 15 was then cast into films on the Indium–Tin–Oxide (ITO)/glass substrate (see ESI†) and charged through corona poling to test its nonlinear behaviour in the presence of an electric field.

**Fig. 2:**
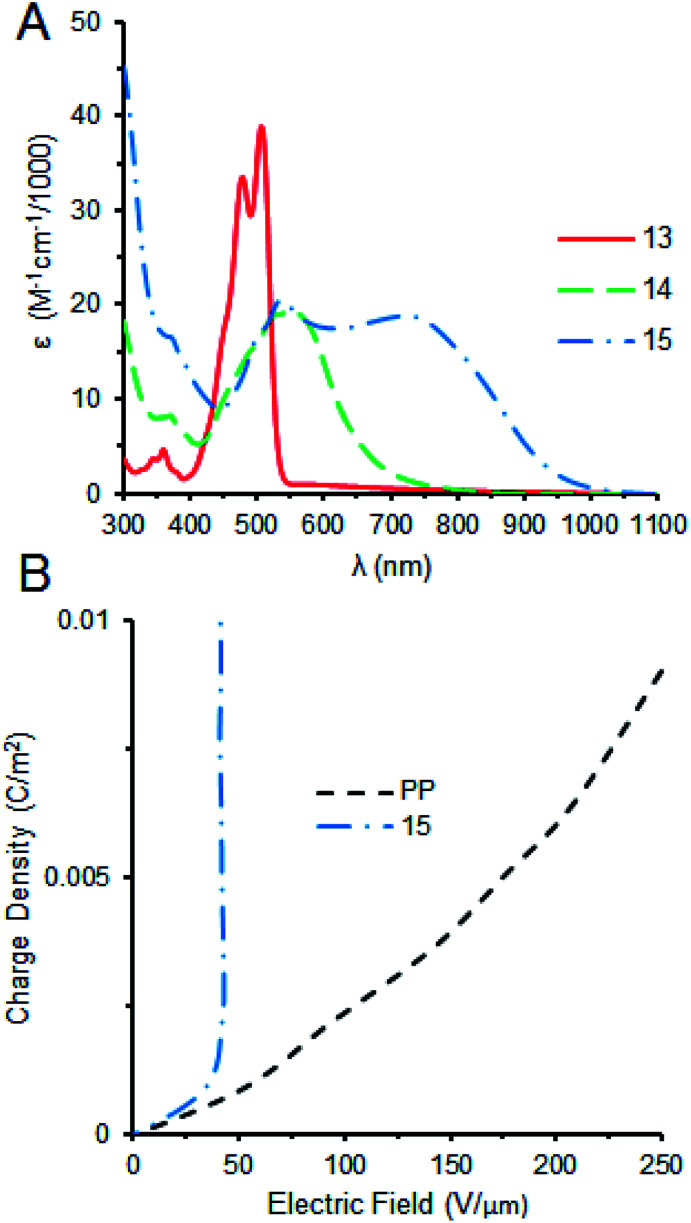
(A) UV-vis absorption spectra of intermediates 13 (solid red), and 14 (dashed green), and final perylene-bisbenzimidazole 15 (dash-dot blue) (0.05 mg mL^−1^ in CHCl_3_). (B) Corona charging capacitor experiment of films cast from polypropylene (PP, dash black) and 15 (dash-dot blue).

In corona poling, a sharp corona tip is charged up to several kilovolts until the electric breakdown of surrounding atmosphere occurs and the positive or negative ions are deposited on the film surface.^11^ With this approach, a large electric field necessary to study the nonlinear response of the material is achieved. Once charge is deposited we used a Kelvin probe to measure the surface potential and its dependence on the amount of deposited charges, which can be determined by measuring the current from the bottom ITO electrode to the ground. This current is directly proportional to the voltage of the corona electrode, as expected, and is controlled by the corona current setting. It is then a simple calculation to convert the time under the corona charge deposition into the charge density on the top of the film. In our experiment, we use the current of 10^−5^ A and the area of the film is 11.5 × 6 cm^2^.

The results of the corona experiment are shown in Fig. 2B for the 2.165 μm thickness film of 15 and 6 μm polypropylene (PP) thin film for the comparison. PP is a common material used as a dielectric in many high voltage capacitors and shows a usual linear relationship between increasing charge density and a growing electric field (Fig. 2B). The dielectric constant of PP is estimated through this approach to be 2.9, close to the tabulated value.^12^ When 15 is exposed to the same conditions, we observe a nonlinear voltage saturation at an electric field of 40 V μm^−1^. We can conclude that in such nonlinear regime the deposition of additional charge leads to the increase of the polarization, not to the increase of the electric field. It should be emphasized that in PP films the electric breakdown occurs after about 10 seconds of the charge deposition, while 15 can sustain up to 5 hours at the same conditions. Correspondingly, the energy stored in the capacitor based on our molecule would be larger than that of the polypropylene-based capacitor by several orders of the magnitude.

## Conclusions

In conclusion, we report the synthesis and characterization of a novel perylene bisbenzimidazole with electron-accepting and donating functional groups. The extended conjugation of 15 is supported by UV-vis absorption measurements. Molecular structure was confirmed through mass spectrometry and IR spectroscopy. Charging measurements taken by a Corona Kelvin probe demonstrate that this material has a nonlinear dielectric response to an increasing electric field with voltage saturation at low electric field strength of 40 V μm^−1^, making it a potent dielectric material for energy storage in a capacitor. Further electric studies on this material are ongoing.

## Conflicts of interest

There are no conflicts to declare.

## Supplementary Material

RA-009-C8RA08873J-s001
