# Supplementary material for: Perylene bisbenzimidazole nonlinear dielectric material for energy storage
Source: RSC Adv. 2019 Jan 2;9(1):361–4. doi: 10.1039/c8ra08873j (PMC9059328; doi:10.1039/c8ra08873j)
Supplement: RA-009-C8RA08873J-s001 [file RA-009-C8RA08873J-s001.pdf]

Electronic Supplementary Material (ESI) for RSC Advances.
This journal is © The Royal Society of Chemistry 2018

Perylene Bisbenzimidazole Nonlinear Dielectric Material for Energy Storage

SAMUEL J. HEIN,[a] CARINE EDDER,[a] MARTA KOWALCZYK,[a,b] ANDREY BORZENKO,[a] LEV MOUROKH,[c] PAVEL LAZAREV[a]

[a] *Capacitor Sciences,1530 O'Brien Rd, Menlo Park, Ca 94025 USA*

[b] Department of Chemistry and Biochemistry, Queens College, City University of New York, 65-30 Kissena Boulevard, Flushing, New York 11367, United States

[c] *1 Physics Department, Queens College of CUNY, 65-30 Kissena Blvd., Flushing, New York 11367, USA*

Supplementary Information

Table of Contents

A. Materials and Instrumentation S2

B. Synthetic Procedures S3

C. NMR Spectroscopy S11

D. UV/Vis Spectroscopy S22

E. DFT Calculations S23

F. Casted Films S26

G. References to Supporting Information S27

A. Materials. Unless otherwise noted, all reagents were purchased from commercial sources and used without further purification. *ortho*-dihydroxybenzene, 1-bromododecane, bis(pinocolato)diboron, Pd(dppf)Cl_2, ammonium sulfide solution, potassium carbonate, diethylamine, benzylamine, ceseium carbonate, palladium hydroxide on carbon, aliquat 336, potassium hydroxide, zinc acetate dihydrate, imidazole, *para*-toluenesulfonic acid and all solvents were purchased from Sigma-Aldrich. 4-bromo-2,6-difluoronitrobenzene was purchased from Combi-Blocks and 2,6-dinitroaniline was purchased from Alfa Aesar. $[(COD)IrOMe]_2$ was purchased by Strem and used without further purification. *N*romosuccinimide was recrystallized prior to use. All reactions were monitored by TLC plastic plates (silica gel 60 F_{254}) purchased from Merk Millipore and all flash chromatography was carried out using a Combi-Flash autocolumn with 40ml/min flow rate and silica gel cartridges with 40g, 80g, 220g, and 330g SiO_2 (230-440 mesh).

Instrumentation. Infrared spectra were recorded on a Thermo Nicolet iS50 with a diamond ART attachment and are uncorrected. UV/vis absorbance spectra were recorded on a Perkin Elmer Lambda 365 spectrometer with a tungsten-halogen and deuterium lamp. High resolution mass spectroscopy were obtained at the University of Massachusetts Mass Spectrometry Core Facility on a JEOL JMS-700 MStation double sector instrument using either EI or FAB mode. Matrix assisted laser desorption ionization time-of-flight (MALDI-TOF) mass spectra were recorded on a Bruker Microflex using a dithranol matrix. CHN Elemental Analysis was obtained from Galbraith Laboratories Inc. NMR spectra were recorded on a JEOL ECZ 400 and Varian Inova 500 from Acorn NMR Inc. All spectra were recorded at ambient temperature. Chemical shifts for ^1H are expressed in parts per million (ppm) relative to tetramethylsilane (δ 0.00 ppm), or $CDCl_3$ (δ 7.26 ppm). Data are reported as follows: chemical shift, multiplicity (s = singlet, d = doublet, t = triplet, dd = doublet of doublets, td = triplet of doublets, m = multiplet, br = broad signal), coupling constant (Hz), and integration. Films of **15** were cast onto a 4 x 8-inch ITO glass slide from a solution of 10 wt.% in chloroform with a Gardco Automatic Drawdown Machine II. The applicator was a size 22 wire-wound bar, speed set to 2 in/sec, and stroke length set to 9 in. The coated glass was dried in ambient conditions overnight before testing.

B: Synthetic Procedures

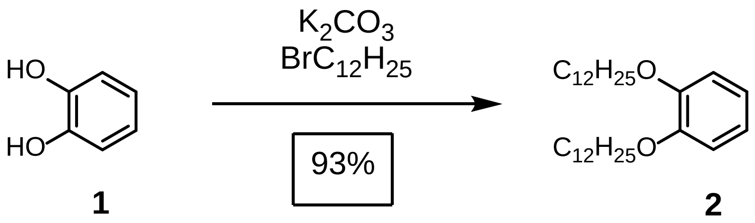

Synthesis of **2**: *ortho*-Dihydroxybenzene (20.01 g, 181.7 mmol, 1 equiv) and K_2CO_3 (75.19 g, 544.0 mmol, 3 equiv) were added to an oven dried flask and purged with vacuum and backfilled with N_2 before being dissolved in DMF (180 mL) and stirred at room temperature for 10 min. Bromododecane (100 mL, 106 g, 428 mmol, 2.3 equiv) was added to this mixture in one portion before being placed in a preheated oil bath set to 100 °C and allowed to stir for 18
hours. When the reaction was complete it was removed from the oil bath and allowed to cool to room temperature in ambient air over 1 hour. Hydrochloric acid (250 mL, 2M) was slowly added to the reaction mixture until the aqueous layer was acidified and the product was extracted with EtOAc (3 x 300 mL). The organic fractions were washed again with H_2O (200 mL). The organic layer was collected and dried under Na_2SO_4, filtered, and the solvent was removed under reduced pressure. The crude material was redissolved in a minimum amount of CH_2Cl_2 (40 mL) and precipitated into MeOH (1000 mL). The white solid was allowed to sit for 1 hour before being filtered to isolate **J232** (75.10 g, 93%). %). ^{1}H and ^{13}C NMR match a previous report.[1]

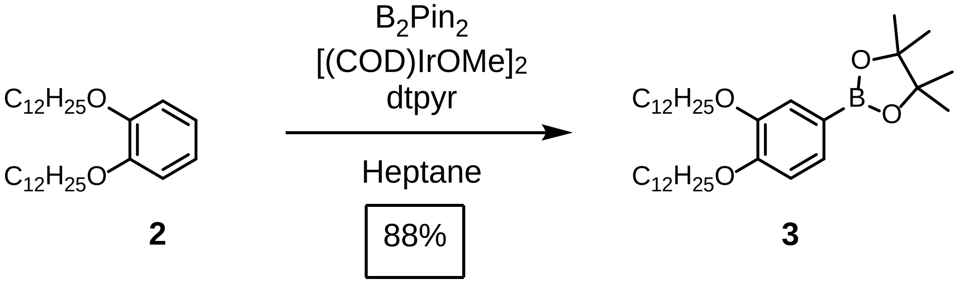

Synthesis of **3**: **2** (30.03 g, 67.15 mmol, 1 equiv) was transferred to a reaction vessel and placed in an oil bath set to 80 °C until melted and placed under vacuum for 15 min to remove any trace MeOH from the previous step. The reaction flask was then removed from heat and allowed to cool to room temperatureover 30 min in air to solidify and backfilled with N_2. To this was added B_2Pin_2 (20.17 g, 80.60 mmol, 1.2 equiv), $[(COD)IrOMe]_2$ (1.088 g, 1.67 mmol, 0.025 equiv), di-*t*-butyldipyridine (0.855 g, 3.36 mmol, 0.05 equiv) and the mixture was evacuated and backfilled with N_2 3 times. In a separate flask, heptane (67 mL) was bubbled with N_2 for 15 min before being added to the reaction flask under an N_2 atmosphere and placed into a preheated 80 °C oil bath. After 16 h, the reaction was removed from the oil bath and allowed to cool to rt. The crude mixture was poured directly onto a SiO_2 plug and eluted with Hexanes/EtOAc (9:1). The solvent was removed to give **3** as a slightly amber oil that slowly solidified over 2 days (33.788 g, 88 % yield). ^{1}H and ^{13}C NMR match a previous report.[2]

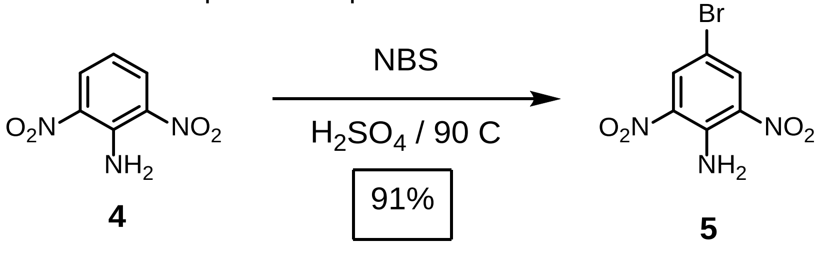

Synthesis of **5**: 2,6-dinitroaniline (10.02 g, 54.71 mmol, 1 equiv) was first dissolved in conc. H_2SO_4 (180 mL) before *N*-bromosuccinimide (13.61 g, 76.48 mmol, 1.4 equiv) was slowly added over the course of 30 min. Once all the NBS had been added, the reaction was placed in a 95 °C oil bath and let to stir for 1 hour. When the reaction was completed it was poured into a 1 L beaker equipped with a magnetic stirbar and filled with crushed ice. Once all the ice had melted, the solid precipitate was filtered off and washed with 400 mL of H_2O. The solid was collected and residual water was removed under reduced pressure overnight. Crude **5** was collected and recrystallized from hexane as a yellow solid (13.073 g, 91%). ^1H NMR (400 MHz, $CDCl_3$): δ 8.65 (s, 2 H), 8.46 (br, 2 H) ppm. ^{13}C NMR (100 MHz, $CDCl_3$) δ 140.6, 136.6, 135.7, 104.9 ppm. FTIR (solid, ATR) 3461, 3348, 3081, 1632, 1509, 1435, 1392, 1352, 1228, 1037, 907, 893, 819, 769, 726, 703, 542, 517, 421 cm^{-1}. HRMS (EI) calcd for $[C_6H_4N_3O_4Br]^+$, 260.9385 found 260.9383.

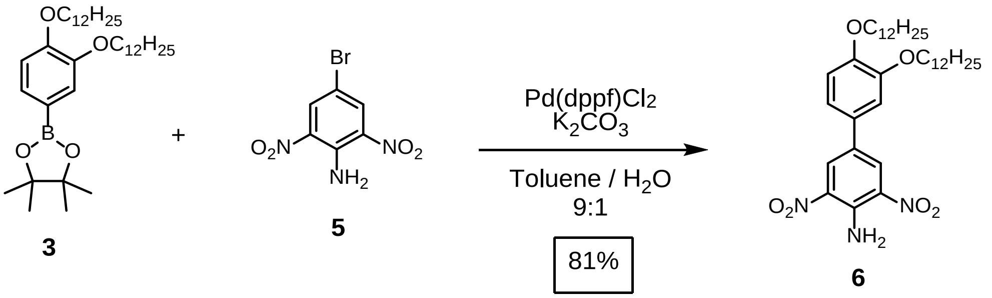

3 **5** **6**

Synthesis of **6**: **5** (7.015 g, 26.77 mmol, 1 equiv), Pd(dppf)Cl_2 (0.975 g, 1.33 mmol, 0.05 equiv), K_2CO_3 (7.384 g, 53.43 mmol, 2 equiv), and **3** (16.93 g, 29.57 mmol, 1.1 equiv) were added to 250 mL round bottom flask. This mixture was then evacuated and backfilled with N_2 3 times. In a separate flask, a mixture of toluene (81 mL) and H_2O (9 mL) was bubbled with N_2 for 30 min. This degassed solvent was then added to the reaction flask under an N_2 atmosphere via canulae and placed into a preheated 100 °C oil bath and was monitored by TLC analysis. After 3 h, the reaction was removed from the oil bath and allowed to cool to rt before being washed with 2M HCl (~100 mL) and extracted using EtOAc (2 x 100 mL). The reaction forms a very bad emulsion at this stage. The emulsion was collected and separated into 5 parts and EtOAc (100 mL) and a saturated brine solution (100 mL) was used to separate the more dilute fractions. The organic layers were collected, dried with Na_2SO_4, filtered, and the solvent was removed under reduced pressure. The crude solid was dissolved in a minimum amount of CH_2Cl_2 (100 mL) and precipitated into 500 mL of MeOH. The solid was filtered and washed with 400 mL of MeOH and dried under reduced pressure overnight. **6** was collected as a red solid (13.748 g, 81%). ^1H NMR (400 MHz, $CDCl_3$): δ 8.73 (s, 2 H), 8.48 (br, 2 H), 7.09 (dd, J = 2.08, 8.40 Hz, 1 H), 7.05 (d, J = 2.08 Hz, 1 H), 6.96, (d, J = 8.40 Hz, 1 H), 4.10-4.01 (m, 4 H), 1.91 – 1.78 (m, 4 H), 1.54 – 1.43 (m, 4 H), 1.26 (s, 32 H), 0.90 – 0.85 (m, 6 H) ppm. ^{13}C NMR (125 MHz, $CDCl_3$) δ 149.9, 140.3, 135.5, 131.8, 129.4, 127.9, 119.2, 114.2, 112.2, 69.8, 69.5, 32.1, 29.9, 29.8, 29.8, 29.8, 29.8, 29.6, 29.6, 29.5, 29.5, 29.4, 26.2, 26.2, 22.8, 14.3 ppm. FTIR (solid, ATR) 3461, 3340, 2918, 2849, 1644, 1588, 1540, 1514, 1466, 1428, 1396, 1349, 1320, 1249, 1212, 1146, 1082, 1032, 902, 856, 799, 770, 721, 606, 559 cm^{-1}. HRMS (EI) calcd for $[C_{36}H_{57}N_3O_6]^+$, 627.4247 found 627.4273.

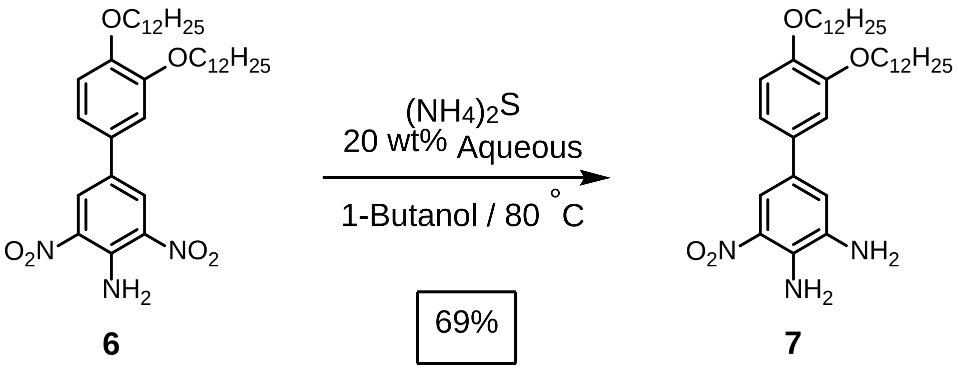

6 **7**

Synthesis of **7**: **6** (5.003 g, 7.913 mmol, 1 equiv) was dissolved in hot butanol (30 mL) in an oil bath 80 °C before adding $(NH_4)_2S$ (20% wt, 5.5 mL, 2 equiv) dropwise. The reaction was allowed to stir at 80 °C for 30 min before adding additional $(NH_4)_2S$ (20% wt, 5.5 mL, 2 equiv) dropwise. The reaction was again allowed to stir at 80 °C for 30 min, after which TLC showed full consumption of starting material. The reaction was removed from the oil bath and allowed to cool to room temperature where it was poured into 200 mL of MeOH with vigorous stirring. The solid was then filtered to give **7** as a red-violet solid (3.261 g, 69%). ^{1}H NMR (400 MHz, $CDCl_3$): δ 7.91 (d, *J* = 1.9 Hz, 1 H),7.15 (d, *J* = 1.9 Hz, 1 H), 7.08-7.02 (m, 2 H), 6.92 (d, J = 9.2 Hz, 1 H), 5.97 (br, 2H) 4.90 – 3.99 (m, 4 H), 3.52 (br, 2 H), 1.90 – 1.75 (m, 4 H), 1.54 – 1.42 (m, 4 H), 1.27 (s, 32 H), 0.90 – 0.85 (m, 6 H) ppm. ^{13}C NMR (125 MHz, $CDCl_3$) δ 149.6, 149.0, 135.9, 135.6, 133.7, 132.4, 130.1, 120.9, 119.1, 115.4, 114.3, 112.6, 69.7, 69.5, 32.1, 29.9, 29.9, 29.8, 29.8, 29.8, 29.8, 29.6, 29.6, 29.6, 29.5, 29.5, 26.2, 26.2, 22.8, 14.3 ppm. FTIR (solid, ATR) 3350, 2917, 2849, 1631, 1587, 1514, 1467, 1427, 1391, 1335, 1313, 1236, 1144, 1072, 1032, 997, 912, 842, 804, 762, 721, 609, 460, 406 cm^{-1}. HRMS (EI) calcd for $[C_{36}H_{59}N_3O_4]^+$, 597.4506 found 597.4500.

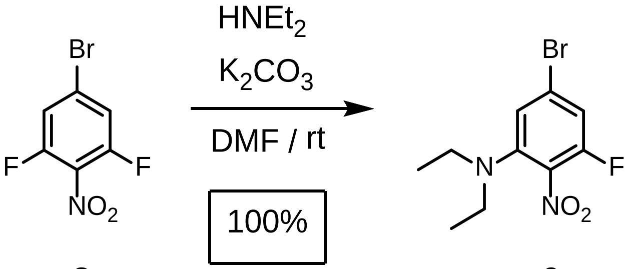

8 **9**

Synthesis of **9**: 4-Bromo-2,6-difluoronitrobenzene (25.01 g, 105.1 mmol) and K_2CO_3 (29.01 g, 210.1 mmol) were dissolved in DMF (260 mL). To this reaction mixture Et_2NH (11.4 mL, 8.07 g, 19.3 mmol, 1.05 mmol) was added and the mixture was allowed to stir at room temperature for 3 hours. Once the reaction was completed, the solution was washed with 500 mL of H_2O and extracted with EtOAc (3 x 200 mL). The organic fractions were combined and washed with additional H_2O (3 x 200 mL) then washed with brine (3 x 100 mL), dried over Na_2SO_4, and the solvent removed under reduced pressure. The crude product isolated as a yellow oil that was used without further purification (31.39 g, 100%). ^{1}H NMR (400 MHz, $CDCl_3$): δ 6.98 (t, *J* = 1.7 Hz, 1 H), 6.88 (dd, *J* = 1.9, 8.84 Hz, 1 H), 3.14 (q, *J* = 7.1 Hz, 4 H), 1.09 (t, *J* = 7.2 Hz, 6 H) ppm. ^{13}C NMR (100 MHz, $CDCl_3$) δ 155.0 (*J* = 259.1 Hz), 145.5, 133.2 (*J* = 15.2 Hz), 124.7 (*J* = 12.3 Hz), 120.0 (*J* = 3.1 Hz), 111.3 (*J* = 22.9 Hz), 46.7, 12.7 ppm. ^{19}F NMR (375 MHz, $CDCl_3$) δ -121.1 (d, *J* = 8.9 Hz, 1F) ppm. FTIR (solid, ATR) 2976, 1740, 1596, 1566, 1527, 1467, 124,

1358, 1263, 1225, 1197, 1146, 1122, 1062, 950, 926, 884, 841, 804, 713, 592, 555, 529, 476 cm^{-1}. HRMS (EI) calcd for $[C_{10}H_{12}N_2O_2BrF]^+$, 290.0066 found 290.0075.

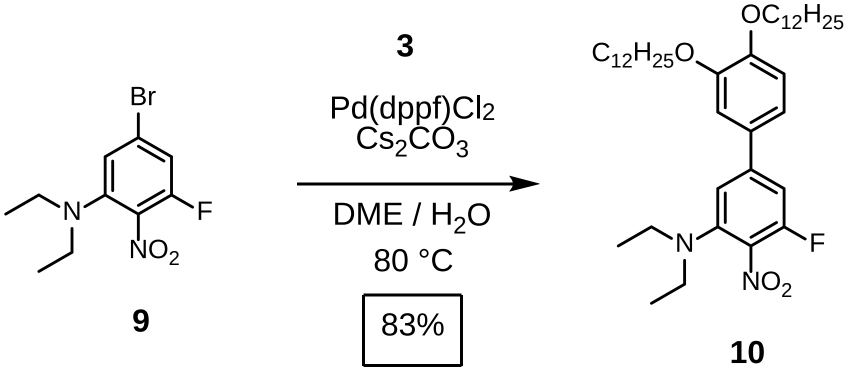

Synthesis of **10**: **3** (87.06 g, 151.9 mmol, 1.1 equiv), Cs_2CO_3 (89.98 g, 325.8 mmol, 2 equiv), and $Pd(dppf)Cl_2$ (5.033 g, 6.87 mmol, 0.05 equiv) were added to a reaction flask and purged with N_2 3 times before adding a degassed solution of **9** (40.00 g, 137.4 mmol, 1 equiv) in DME (330 mL) that had been sparging under a stream of N_2 for 20 min. In a separate flask, H_2O (110 mL) was sparged for 20 min before being added to the reaction flask under a N_2 atmosphere then placed into an 80 °C oil bath. This reaction was allowed to stir at temperature for 5 hours and was monitored by TLC. Once TLC showed consumption of **9**, the solution was acidified with HCl (300 mL, 2M) and extracted with EtOAc (3 x 200 mL). The organic layer was washed once with brine (100 mL), dried over Na_2SO_4 and the solvent was removed under reduced pressure. The crude product was triturated in 300 mL of MeOH to give **10** as a yellow solid and was recrystallized from hot ethanol (74.49 g, 83%).^{1}H NMR (400 MHz, $CDCl_3$): δ 7.06 (dd, *J* = 2.3, 8.3 Hz, 1 H), 7.03 (d, *J* = 2.3 Hz, 1 H), 7.01 (s, 1 H), 6.94 (d, *J* = 8.3 Hz, 1H), 6.93 (dd, *J* = 1.7, 10.5, 1H), 4.05 (q, *J* = 6.6 Hz, 4 H), 3.17 (q, *J* = 7.0, 4 H), 1.85 (p, *J* = 6.7 Hz, 4 H), 1.49 (m, 4 H), 1.40 – 1.27 (m, 32 H), 1.11 (t, *J* = 7.1 Hz, 6 H), 0.88 (m, 6 H) ppm. ^{13}C NMR (100 MHz, $CDCl_3$) δ 155.1 (*J* = 254.3 Hz), 149.9 (*J* = 71.7 Hz), 145.3 (*J* = 1.8 Hz), 144.6 (*J* = 9.5 Hz), 134.0 (*J* = 14.5 Hz), 131.0 (*J* = 2.2 Hz), 120.1, 115.9 (*J* = 2.1 Hz), 113.6 (*J* = 66.2 Hz), 107.2 (*J* = 20.1 Hz), 69.7 (*J* = 44.1 Hz), 47.2, 32.1, 29.9, 29.8, 29.8, 29.6, 29.6, 29.5, 29.5, 29.4, 26.2, 22.8, 14.3, 12.9 ppm. ^{19}F NMR (375 MHz, $CDCl_3$) δ -122.9 (d, *J* = 10.7 Hz, 1F) ppm. FTIR (solid, ATR) 2917, 2849, 1738, 1610, 1573, 1495, 1467, 1379, 1360, 1259, 1235, 1192, 1145, 1119, 1062, 997, 957, 916, 843, 816, 802, 721, 640, 589, 531, 457, 419 cm^{-1}. HRMS (EI) calcd for $[C_{40}H_{65}N_2O_4F]^+$, 656.4928 found 656.4901.

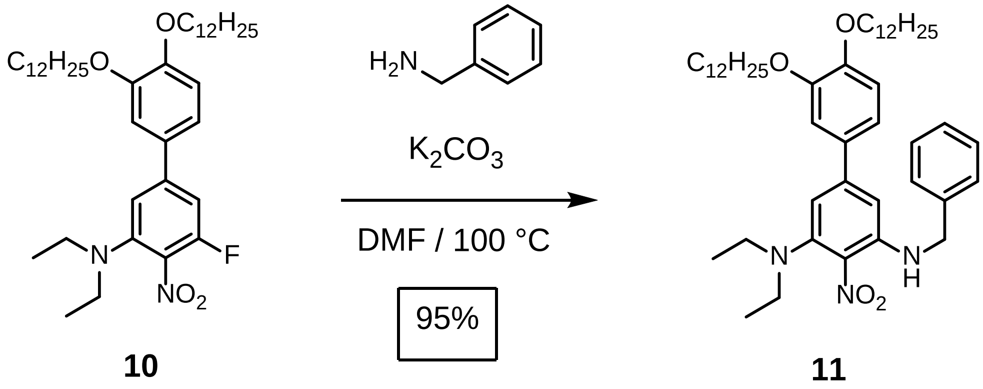

Synthesis of **11**: **10** (10.08 g, 15.34 mmol), K_2CO_3 (4.283 g, 30.98 mmol, 2 equiv), and benzylamine (6.7 mL, 60.89 mmol, 4 equiv) were added to DMF (50 mL) and heated to 100 °C while stirring. When the reaction was complete it was removed from the oil bath and allowed to cool to room temperature in ambient air. Then, the reaction was washed with H_2O (100 mL), and extracted with Et_2O (3 x 50 mL). The organic fractions were combined, dried over Na_2SO_4, and the solvent removed under reduced pressure. Purification by SiO_2 plug (10% EtOAc in Hexanes) afforded **11** as a red oil (11.156 g, 95%). that was used at the next step without further purification. ^1H NMR (400 MHz, $CDCl_3$): δ ppm. ^{13}C NMR (100 MHz, $CDCl_3$) δ ppm. FTIR (solid, ATR) 2922, 2852, 1568, 1512, 1466, 1377, 1357, 1295, 1254, 1142, 1123, 1068, 1028, 848, 804, 755, 697, 665, 459 cm^{-1}. HRMS (EI) calcd for $[C_{47}H_{73}N_3O_4]^+$, 743.5601 found 743.5613.

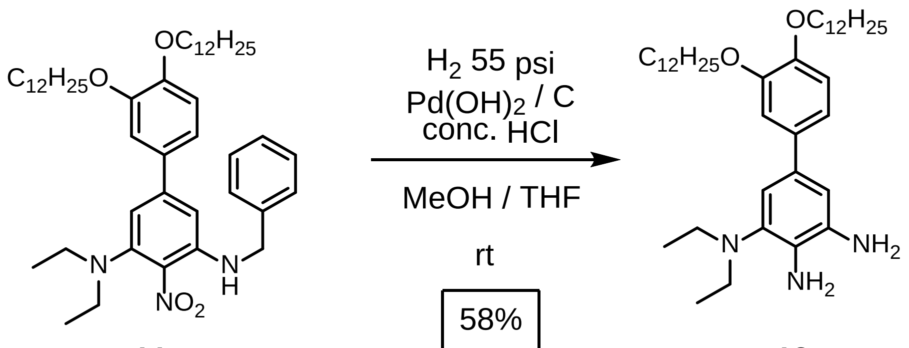

11 **12**

Synthesis of **12**: **11** (5.037 g, 6.769 mmol, 1 equiv) was dissolved in 1:1 MeOH/THF (12 mL) before adding $Pd(OH)_2$ / C (0.942 g, 0.2 equiv, 20 wt %) and concentrated HCl (0.44 mL, 12 M, 4 equiv). This mixture was pressurized with H_2 (55 psi) and stirred for 1 hour. Once complete, the reaction mixture was filtered through a celite plug washed with $NaOH_{(aq)}$ (25 mL, 1M) and extracted with EtOAc (3 x 25 mL). The organic fractions were collected and dried with Na_2SO_4 and the solvent removed under reduced pressure. The residue was then purified SiO_2 chromatography (25 → 50 % EtOAc in Hexanes) to afford **12** as a brownish waxy solid (2.439 g, 58 %). ^1H NMR (400 MHz, $CDCl_3$): δ 7.05 (d, *J* = 2.0 Hz, 1 H), 7.03 (dd, *J* = 2.0, 8.2 Hz, 1 H), 6.91 (d, *J* = 8.3 Hz, 1 H), 6.82 (d, *J* = 1.9 Hz, 1 H), 6.73 (d, *J* = 1.9 Hz, 1 H), 4.06 (t, *J* = 6.5 Hz, 2 H), 4.02 (t, *J* = 6.7 Hz, 2 H), 3.87 (br, 2 H), 3.49 (br, 2H), 2.98 (q, *J* = 6.8 Hz, 4 H), 1.84 (m, 4 H), 1.49 (m, 4 H), 1.36 (m, 4 H), 1.28 (s, 24 H), 1.02 (t, *J* = 7.1 Hz, 6 H), 0.89 (m, 6 H) ppm. ^{13}C NMR (100 MHz, $CDCl_3$) δ 149.3, 148.2, 138.8, 135.3, 135.0, 132.1, 132.0, 119.1, 114.3, 113.1, 113.1, 111.2, 69.6, 69.6, 48.5, 32.1, 29.8, 29.8, 29.6, 29.6, 29.6, 29.5, 26.2, 26.2, 22.8, 14.3, 13.1 ppm. FTIR (solid, ATR) 3401, 3320, 2850, 1640, 1577, 1518, 1492, 1469, 1407, 1380, 1314, 1256, 1234, 1209, 1186, 1137, 1069, 1014, 915, 841, 812, 760, 721, 625, 581, 460 cm^{-1}. HRMS (EI) calcd for $[C_{40}H_{69}N_3O_2]^+$, 623.5390 found 623.5403.

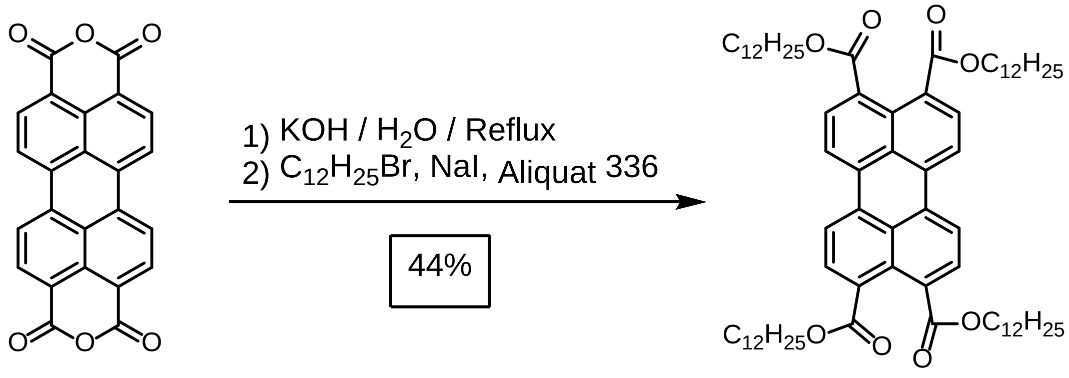

S1

Synthesis of **S1**: Perylene dianhydride (30.01g, 76.49 mmol, 1 equiv) was added to a solution of KOH (17.39 g, 269.6 mmol, 87 wt % pellets, 3.5 equiv) in water (200 mL). This suspension was heated to reflux for 30 min, after which aliquat 336 (15 g, 37 mmol, 0.5 equiv) and NaI (1.152 g, 7.686 mmol, 0.1 equiv) was added to the reaction mixture and stirred vigorously for 10 min. 1-Bromododecane (146 mL, 611.7 mmol, 8 equiv) was then added to the reaction mixture in one portion. The mixture was heated to reflux with vigorous stirring for an additional 2 h. Once the reaction was complete, the mixture was cooled to room temperature and filtered. The crude orange solid was then dissolved in 500 mL of $CHCl_3$ and filtered again to remove a dark red precipitate and the filtrate was concentrated on reduced pressure. The organic solution was then poured into MeOH (1.5 L) and the resulting orange solid was collected by filtration and washed with additional MeOH to afford **S1** (18.2 g, 44%). ^{1}H and ^{13}C NMR match a previous report.[3]

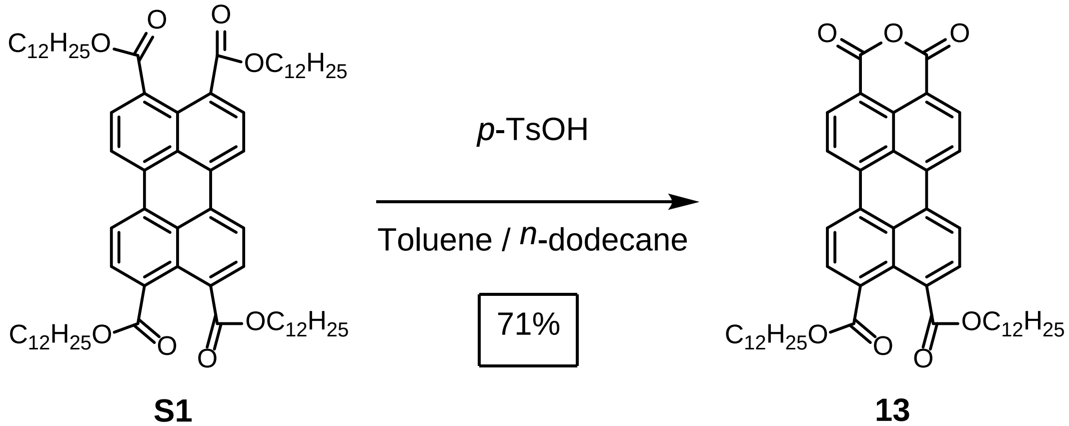

S1 **13**

Synthesis of **13**: **S1** (30.02 g, 27.25 mmol, 1 equiv) was dissolved into a mixture of toluene (15 mL) and *n*-dodecane (75 mL). To this solution, *p*-toluenesulfonic acid monohydrate (5.385 g, 28.31 mmol 1.03 equiv) was added and the reaction mixture was heated to 95 °C for 4 hours. The dark red precipitate was diluted into boiling THF (300 mL) and this solution was precipitated into MeOH (1.2 L). The solid was collected by vacuum filtration and washed with MeOH (500 mL) to afford **13** as a blood-red solid (14.55 g, 71%). ^{1}H and ^{13}C NMR match a previous report.[4]

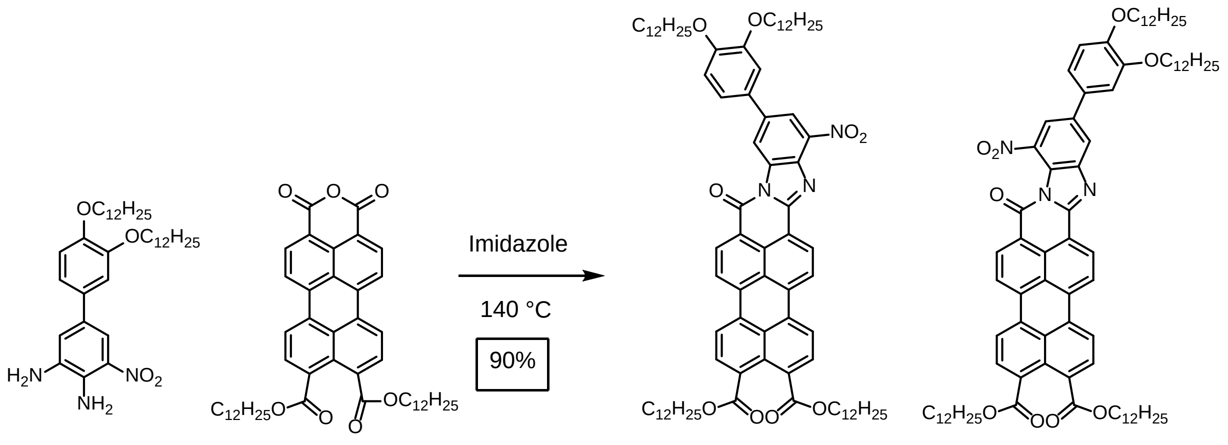

7 **13** **S2**

Synthesis of **S2**: **7** (4.466 g, 7.470 mmol, 1.1 equiv), **13** (5.008 g, 6.704 mmol, 1.0 equiv) and imidazole (31.95 g, 469.3 mmol, 70 equiv) added to a reaction flask and purged with N_2. This mixture was heated to 140 °C for 4 hours after which the mixture was poured into vigorously stirring MeOH (50 mL) where the solid precipitated as a mixture of isomers. **S2** was collected *via* vacuum filtration to give a violet solid (7.949 g, 90%). ^{1}H NMR (400 MHz, $CDCl_3$): δ 9.07 (s), 9.03 (d, *J* = 7.5 Hz), 8.93 (d, *J* = 2.7 Hz), 8.80 (d, *J* = 7.5 Hz), 8.57 (d, *J* = 8.0 Hz), 8.25-8.47 (m), 8.17 (d, *J* = 8.5 Hz), 8.12 (d, *J* = 8.5 Hz), 7.83 (d, J = 8.3 Hz), 7.73 (t, *J* = 7.0 Hz), 7.56 (m), 7.41 (dd, *J* = 4.1, 8.2 Hz) 4.35 (m) 4.17 – 3.99 (m), 1.92 – 1.78 (m), 1.66 – 1.55 (m), 0.87 (m) ppm. Due to low solubility, a ^{13}C NMR spectrum could not be obtained. FTIR (solid, ATR) 2918, 2850, 1772, 1704, 1593, 1515, 1464, 1355, 1301, 1200, 1169, 1149, 1105, 1072, 1026, 1016, 961, 938 cm^{-1}. HRMS (FAB) calcd for $[C_{84}H_{113}N_3O_9]H^+$, 1308.8550 found 1308.8420.

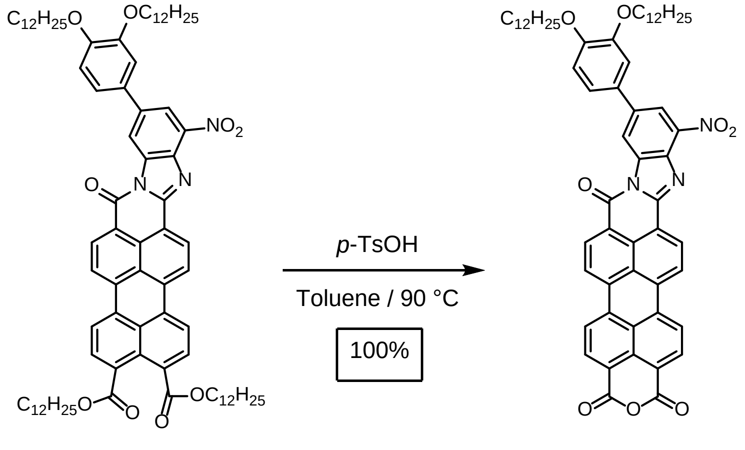

S2 **14**

Synthesis of **14**: **S2** (5.033 g, 3.845 mmol, 1 equiv) was dissolved into toluene (5 mL). To this solution, *p*-toluenesulfonic acid monohydrate (7.314 g, 38.45 mmol, 10 equiv) was added and the reaction mixture was heated to 90 °C for 12 hours. The mixture was diluted in $CHCl_3$ (50 mL) and this solution was precipitated into MeOH (400 mL). The solid was collected by centrifugation and washed with MeOH (200 mL) and was filtered to afford **14** as a purple solid

(3.985 g, 100%). Due to low solubility ^{1}H and ^{13}C NMR spectra could not be obtained. FTIR (solid, ATR) 2920, 2850, 1769, 1701, 1591, 1517, 1464, 1323, 1300, 1250, 1144, 1122, 1009, 962, 850, 806, 792, 783, 736, 680, 568 cm^{-1}. HRMS (FAB) calcd for $[C_{60}H_{63}N_3O_8]H^+$, 954.4688 found 954.4710.

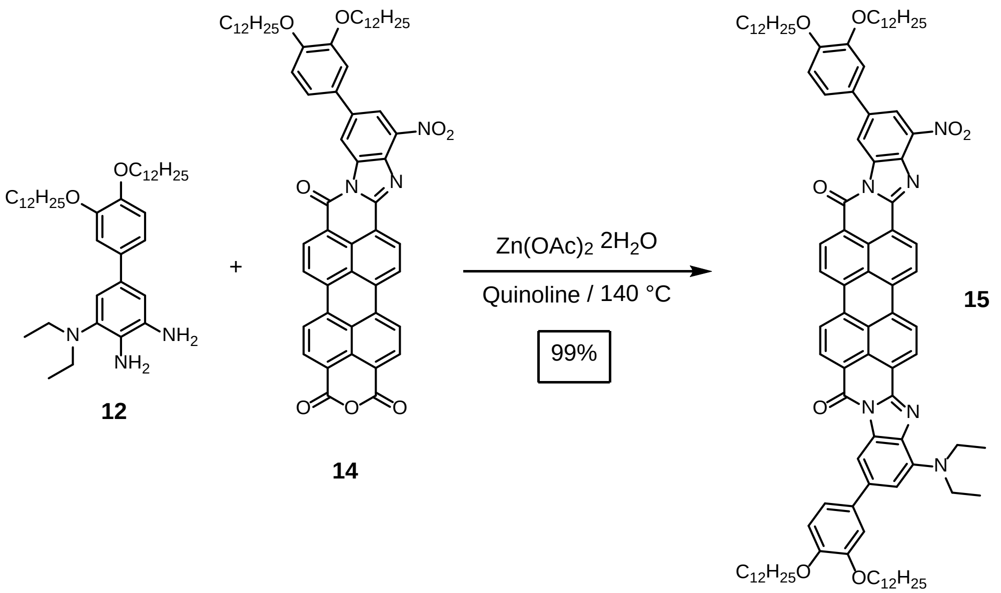

Synthesis of **15**: **12** (1.767 g, 2.831 mmol, 1.3 equiv), **14** (1.995 g, 2.096 mmol, 1 equiv) and $Zn(OAc)_2$ $2H_2O$ (0.384 g, 2.096 mmol, 1 equiv) were added to a reaction flask and purged with N_2. In a separate flask quinoline (7 mL) was sparged under a ^{N2} flow for 15 min and then added to the reaction flask. This mixture was heated to 140 °C and allowed to stir overnight. When the reaction was completed it was poured into MeOH (100 mL) and the solid precipitated. **15** was collected via vacuum filtration as a black solid (3.210 g, 99%). ^{1}H NMR (400 MHz, 10 % dTFA in $CDCl_3$): δ 9.61 (t, *J* = 7.7 Hz), 9.47 (d, 4.18 Hz), 9.31 – 9.38 (m), 9.23 (d, *J* = 7.35 Hz), 9.20 – 9.07 (m), 9.03 (d, *J* = 5.4 Hz), 8.84 (s), 8.22 (s), 8.10 (s), 7.93 (s), 7.88 (s), 7.86 (s), 7.77 (s), 7.59 (d, *J* = 1.7 Hz), 7.44 (dd, *J* = 2.2, 8.3 Hz), 7.40 – 7.34 (m), 7.21 – 7.12 (m), 4.3 – 4.08 (m), 1.98 – 1.76 (m), 1.58 – 1.44 (m), 1.44 – 1.19 (m), 0.92 – 0.82 (m) ppm. ^{13}C NMR spectra could not be obtained due to low solubility. FTIR (solid, ATR) 2920, 2851, 1691, 1593, 1515, 1501, 1465, 1358, 1256, 1189, 1138, 1047, 1015, 978, 921, 835, 804, 769, 742, 721, 607, 501, 444 cm^{-1}. LRMS (MALDI) calcd for $[C_{100}H_{128}N_6O_8]^+$, 1542.0 found 1541.8. Anal. Calcd. For $C_{100}H_{128}N_6O_8$: C, 77.88; H, 8.37; N, 5.45; Found C, 76.13; H, 8.62; N, 5.60.

C: 1D NMR

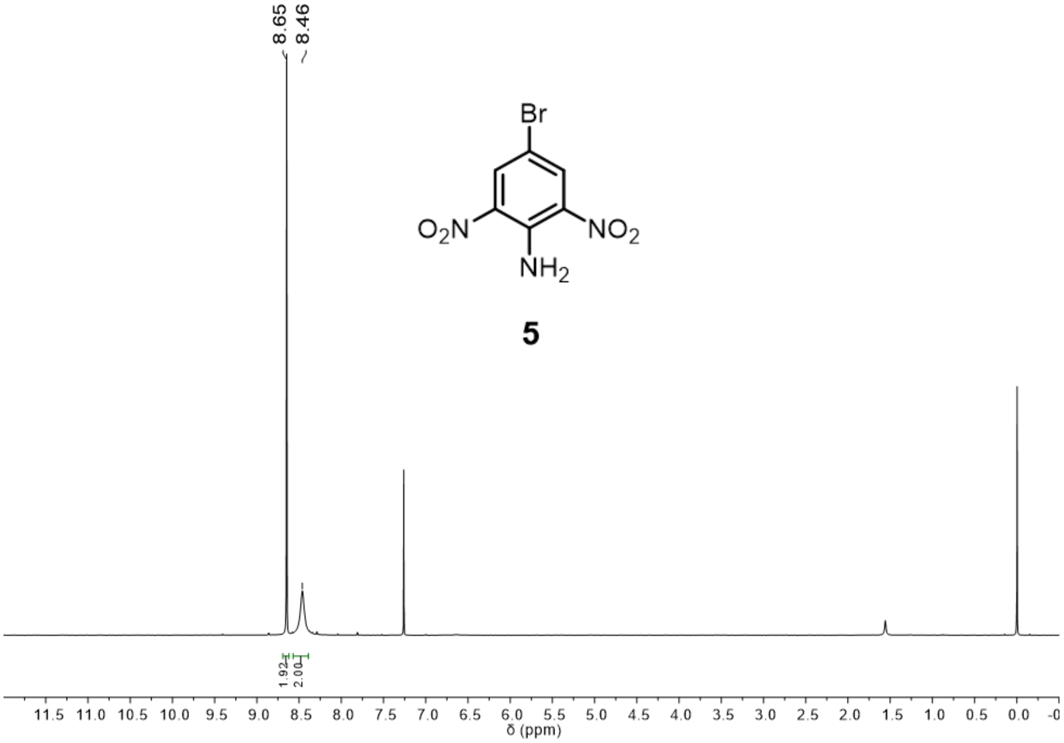

Figure S1. ^{1}H NMR of **5** (400 MHz, $CDCl_3$, 275 K)

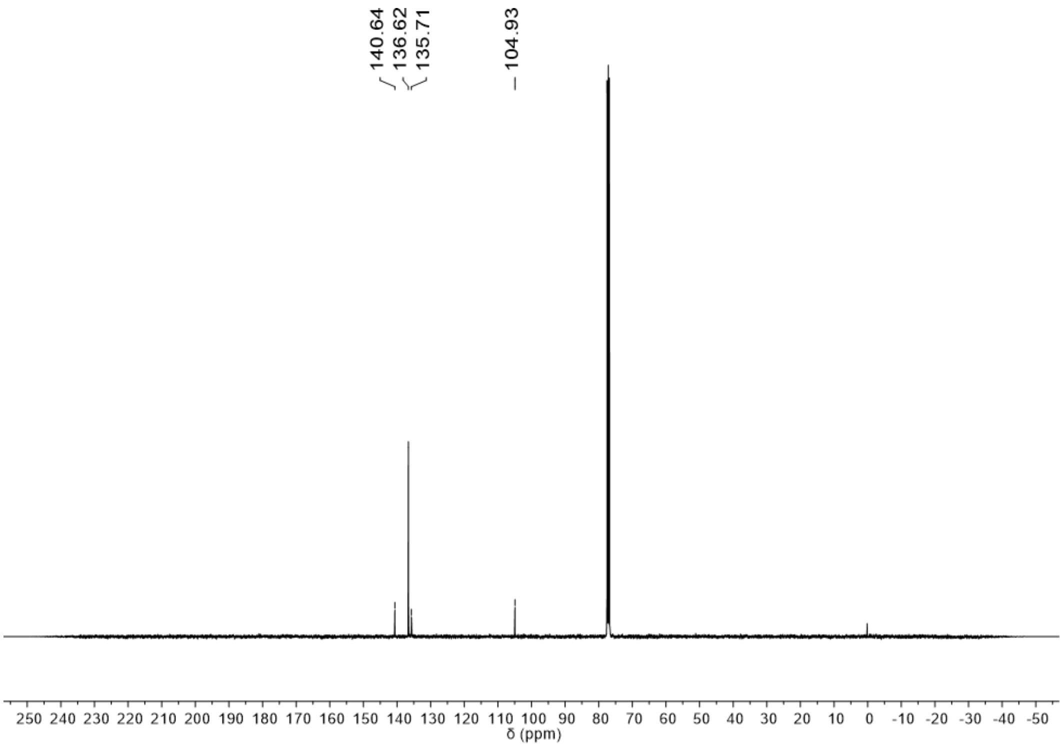

Figure S2. ^{13}C NMR of **5** (100 MHz, $CDCl_3$, 275 K)

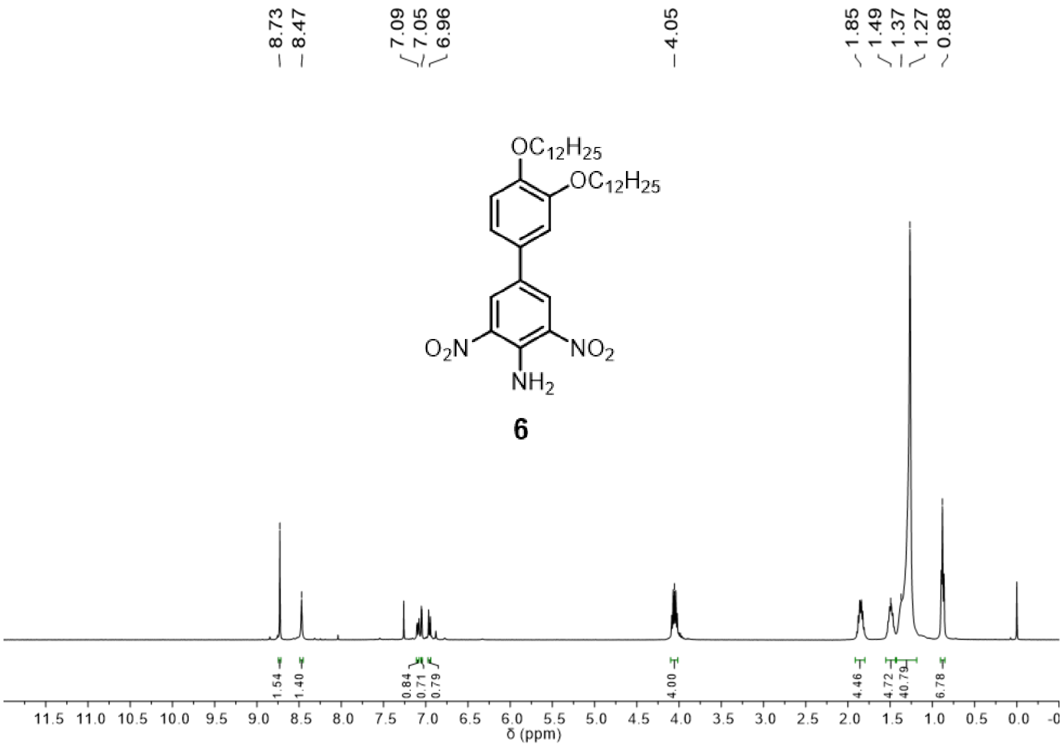

Figure S3. ^{1}H NMR of **6** (400 MHz, $CDCl_3$, 275 K)

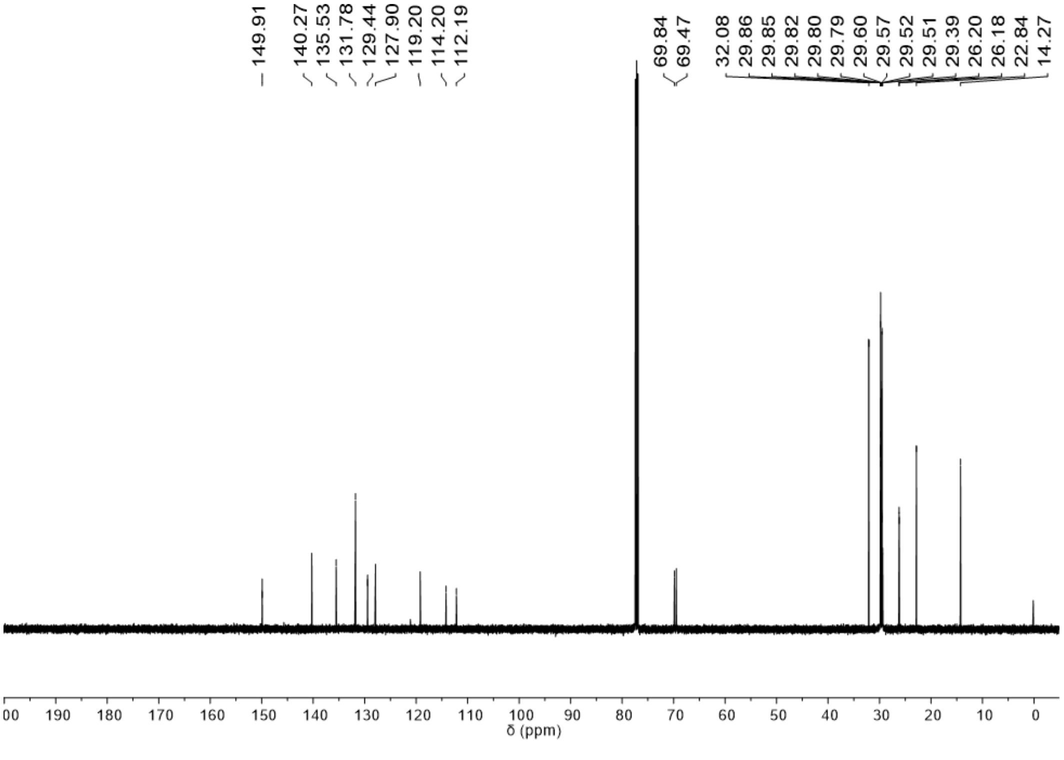

Figure S4. ^{13}C NMR of **6** (100 MHz, $CDCl_3$, 275 K)

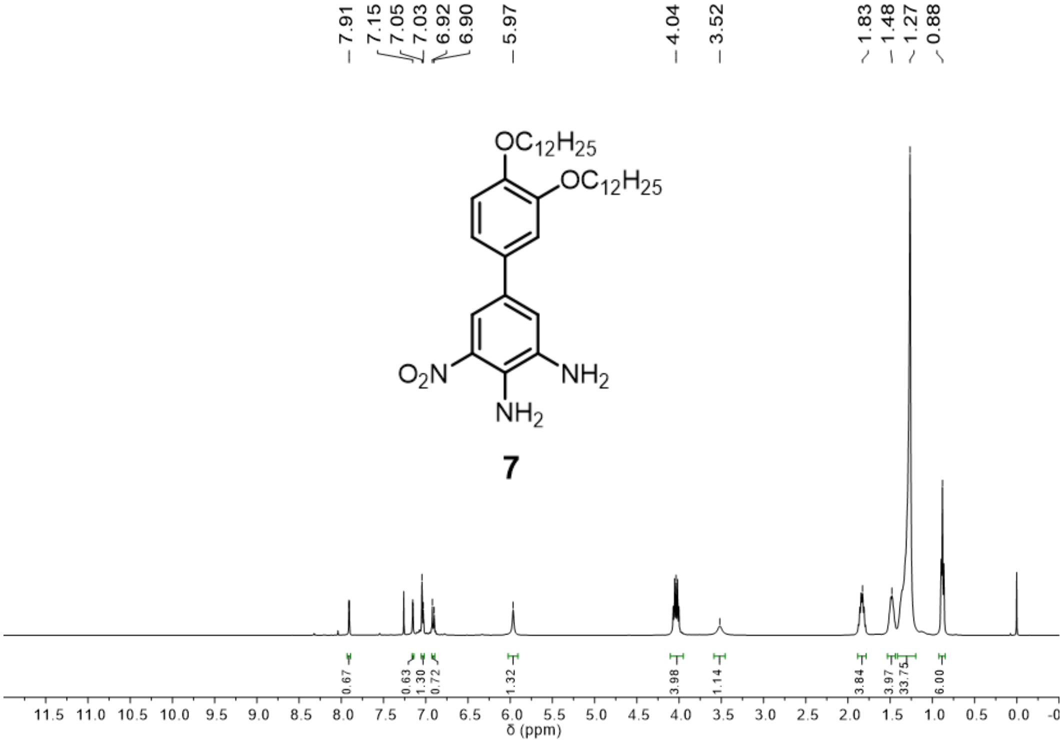

Figure S5. ^{1}H NMR of **7** (400 MHz, $CDCl_3$, 275 K)

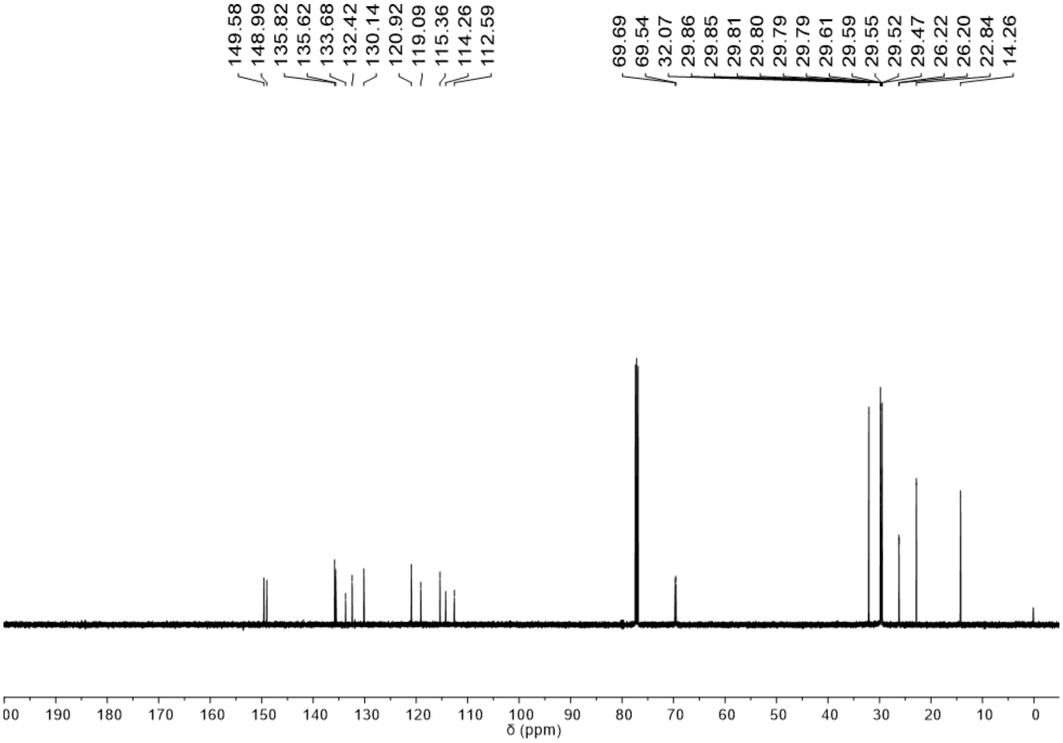

Figure S6. ^{13}C NMR of **7** (100 MHz, $CDCl_3$, 275 K)

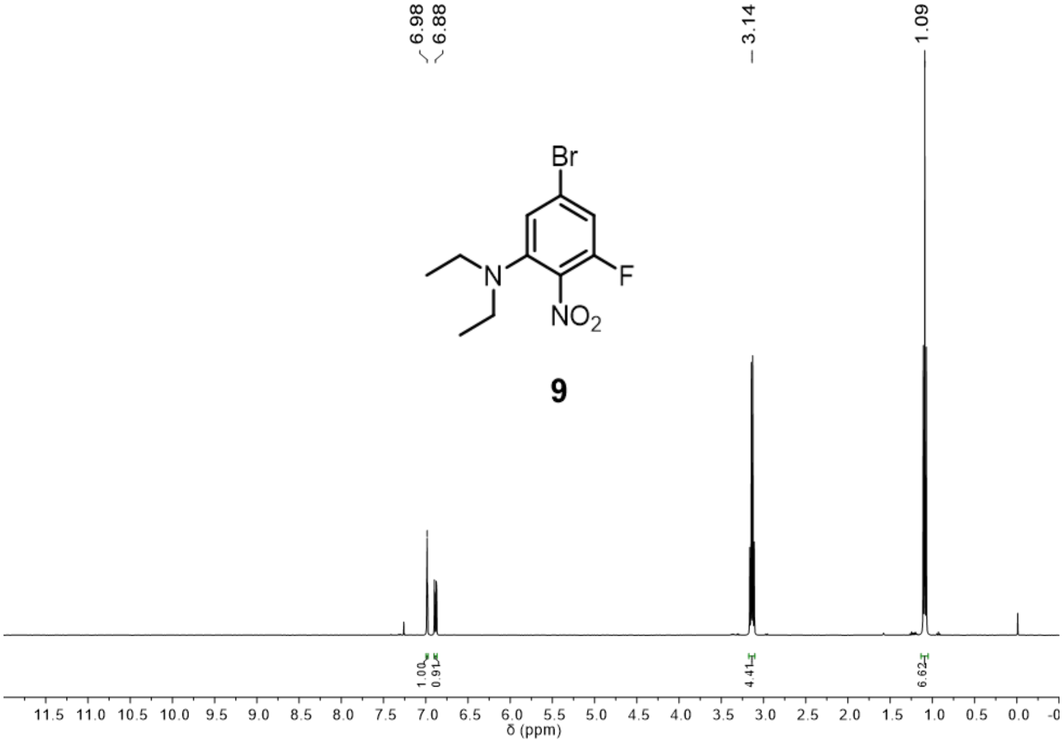

Figure S7. ^{1}H NMR of **9** (400 MHz, $CDCl_3$, 275 K)

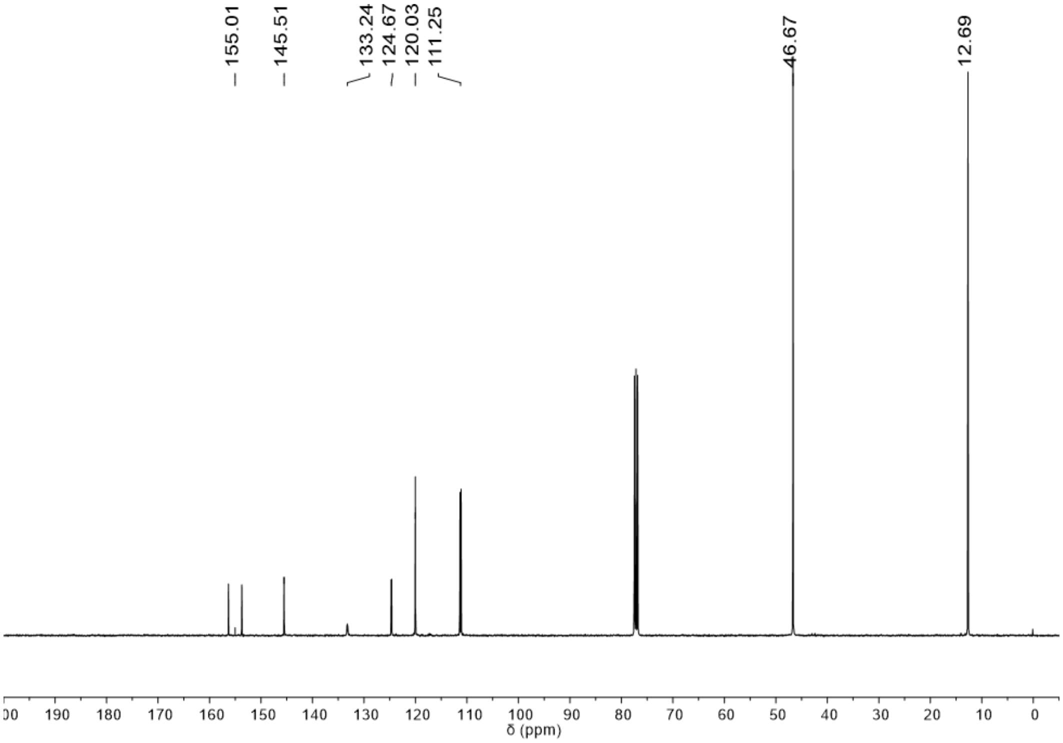

Figure S8. ^{13}C NMR of **9** (100 MHz, $CDCl_3$, 275 K)

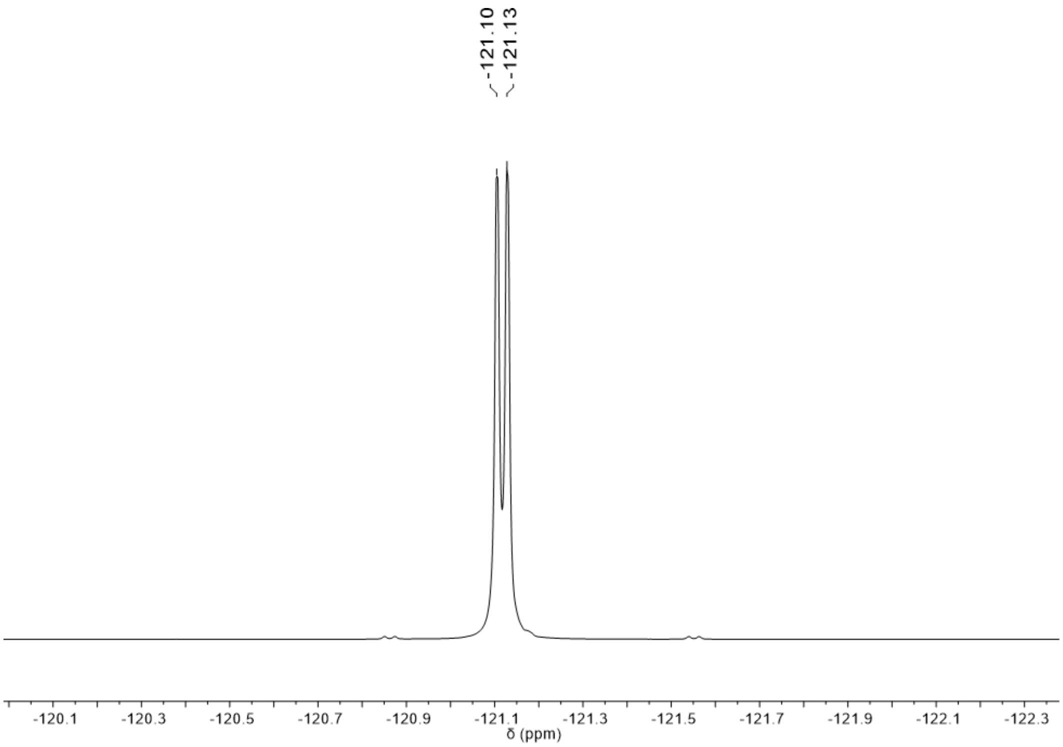

Figure S9. ^{19}F NMR of **9** (375 MHz, $CDCl_3$, 275 K)

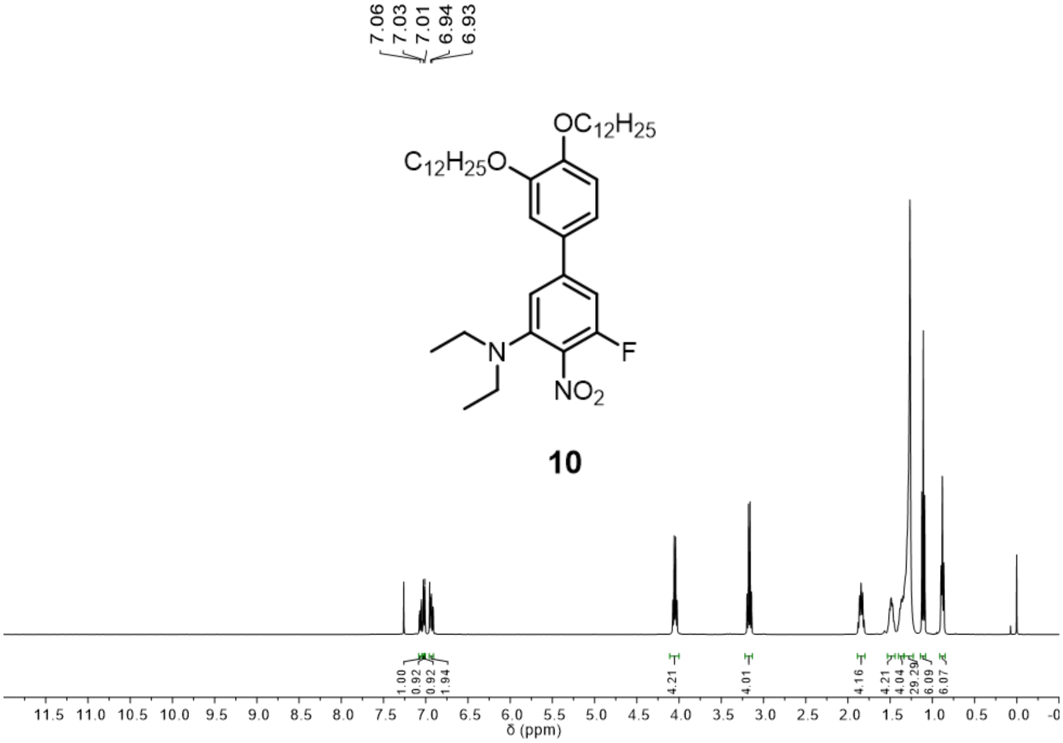

Figure S10. ^{1}H NMR of **10** (400 MHz, $CDCl_3$, 275 K)

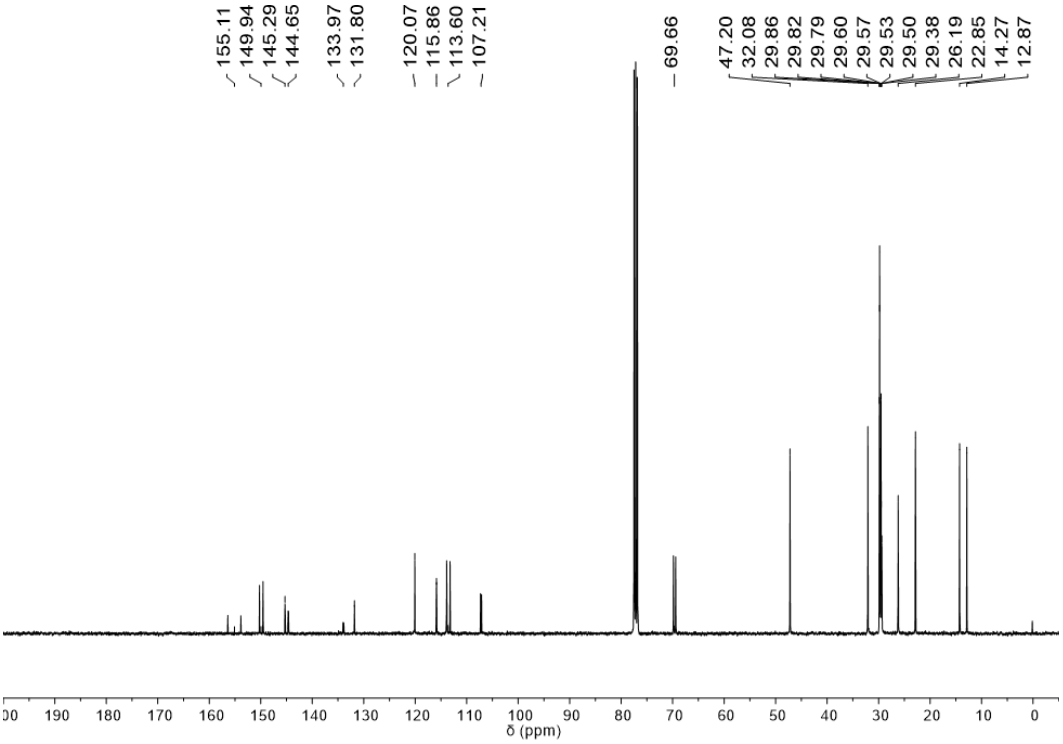

Figure S11. ^{13}C NMR of **10** (100 MHz, $CDCl_3$, 275 K)

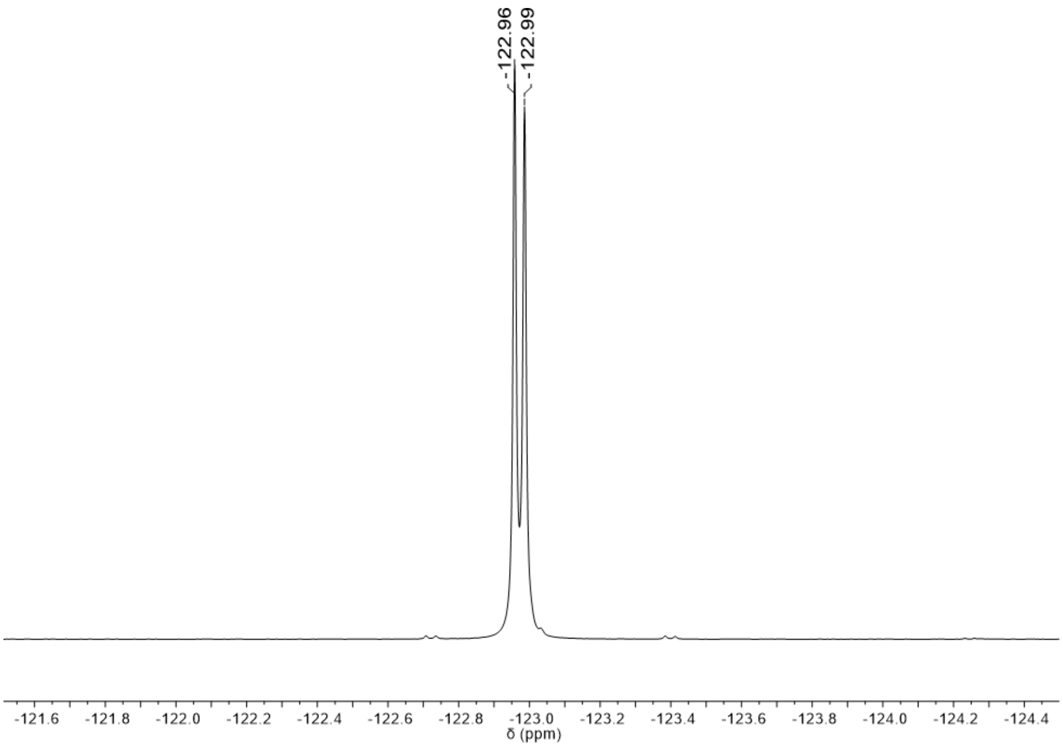

Figure S12. ^{19}F NMR of **10** (375 MHz, $CDCl_3$, 275 K)

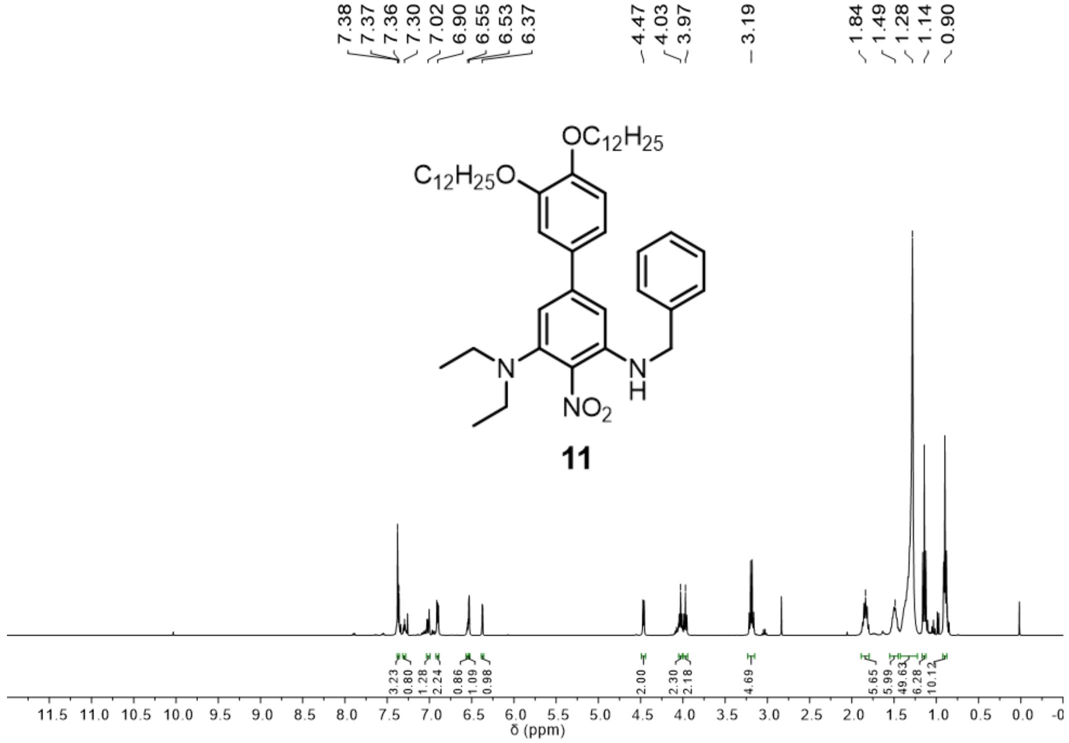

Figure S13. ^{1}H NMR of **11** (400 MHz, $CDCl_3$, 275 K)

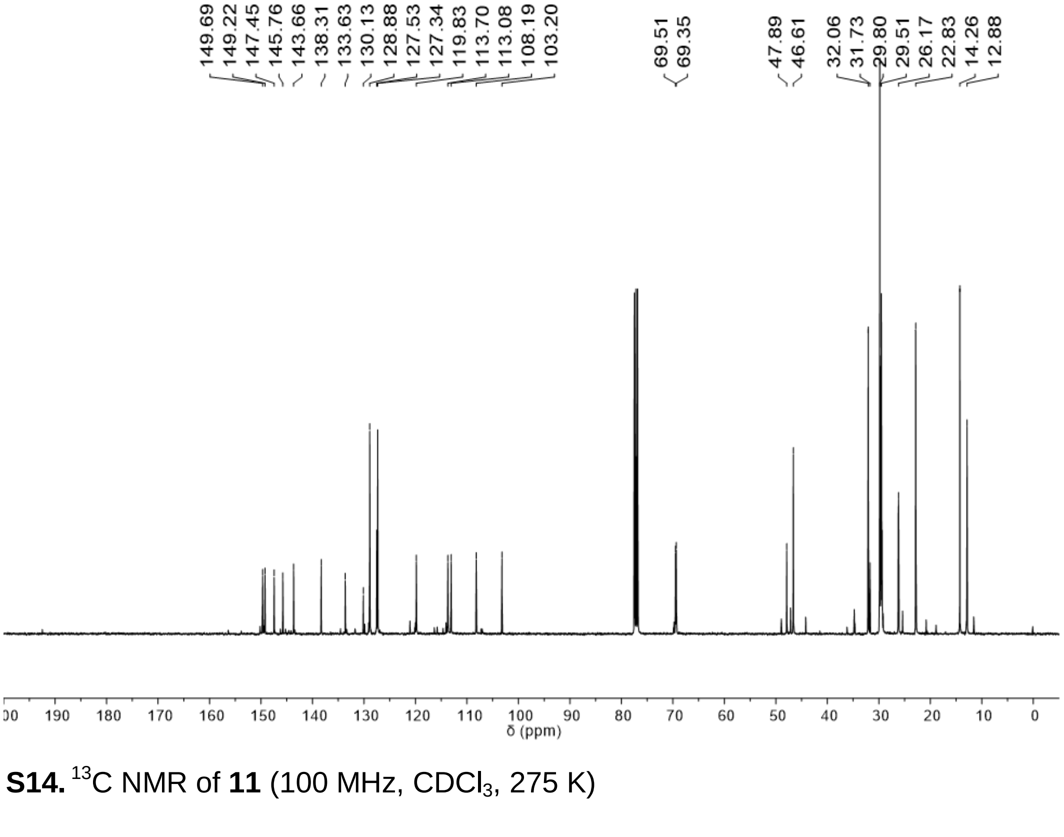

Figure S14. ^{13}C NMR of **11** (100 MHz, $CDCl_3$, 275 K)

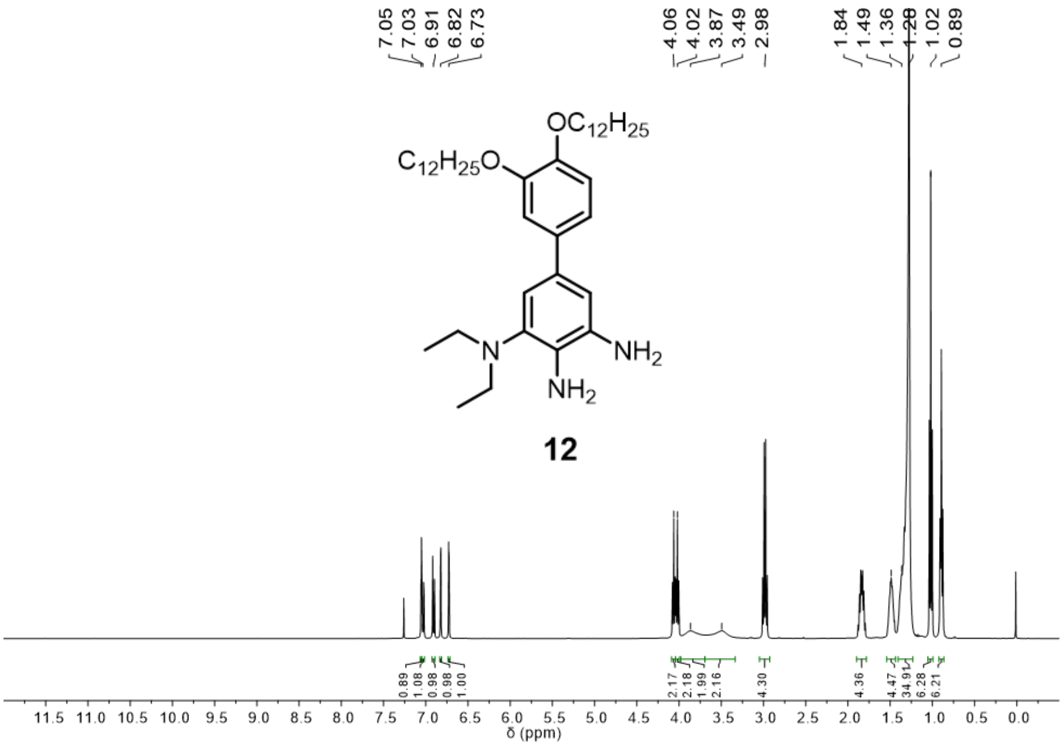

Figure S15. ^{1}H NMR of **12** (400 MHz, $CDCl_3$, 275 K)

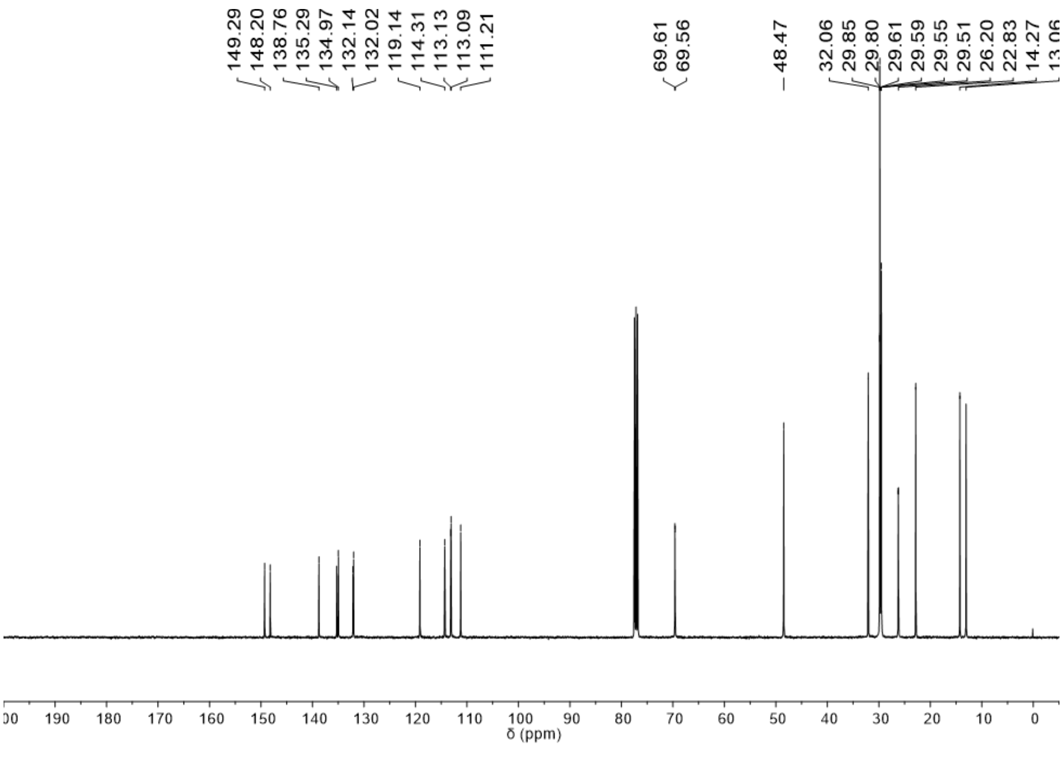

Figure S16. ^{13}C NMR of **12** (100 MHz, $CDCl_3$, 275 K)

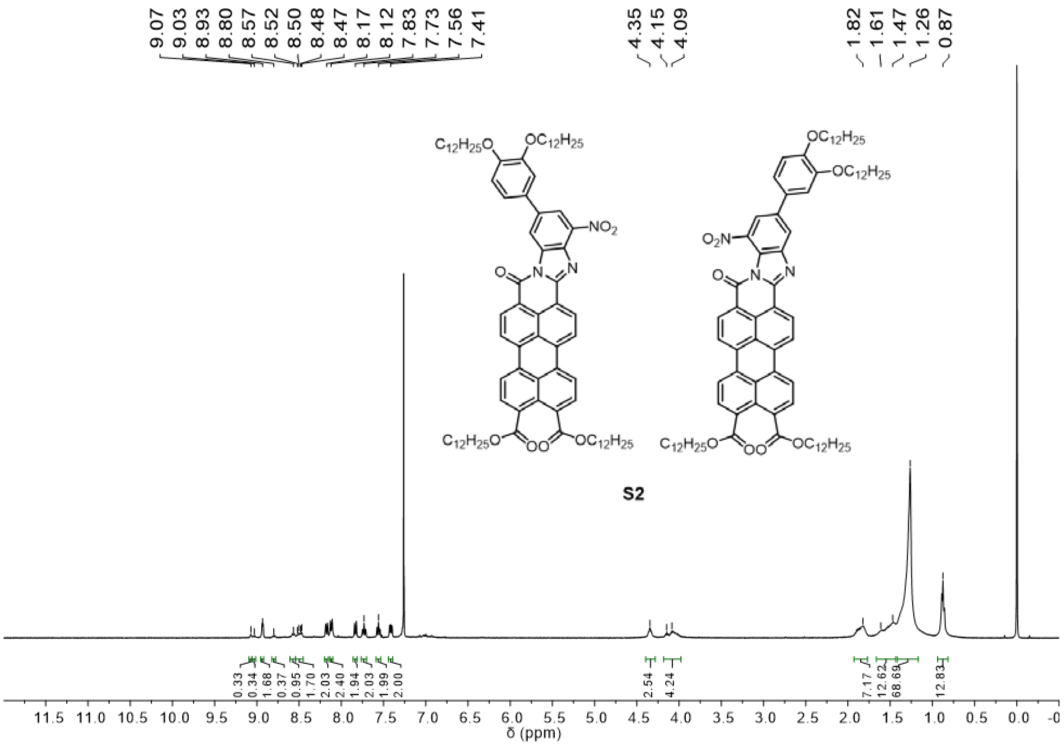

Figure S17. ^{1}H NMR of **S2** (400 MHz, $CDCl_3$, 275 K)

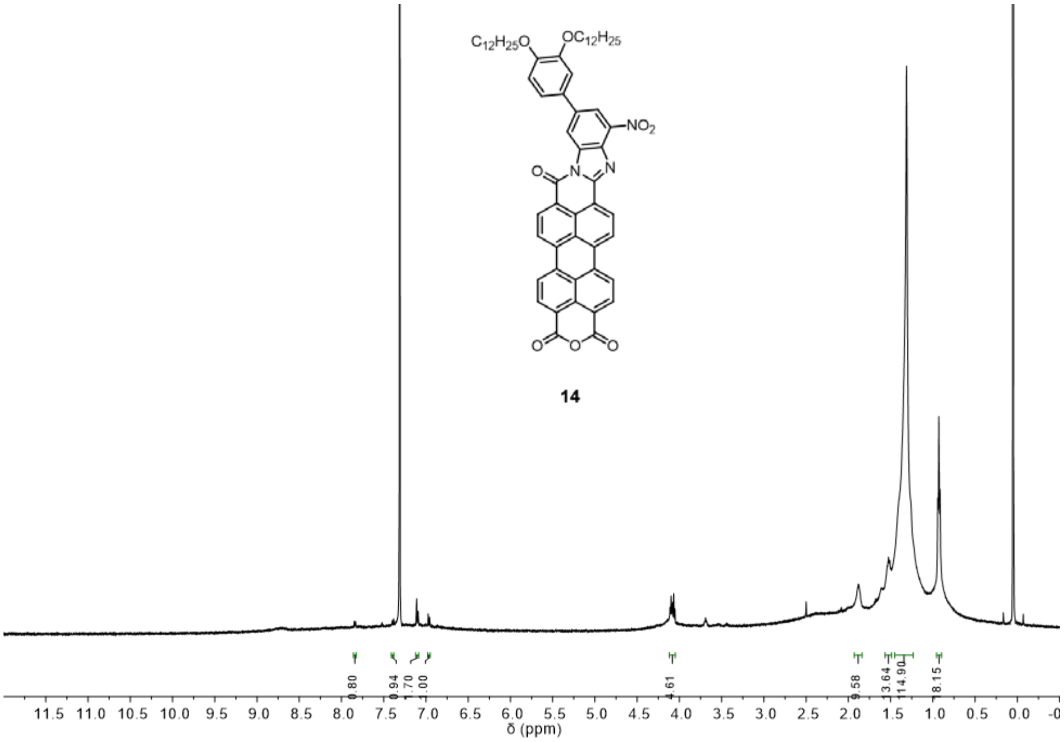

Figure S18. ^{1}H NMR of **14** (400 MHz, $CDCl_3$, 275 K)

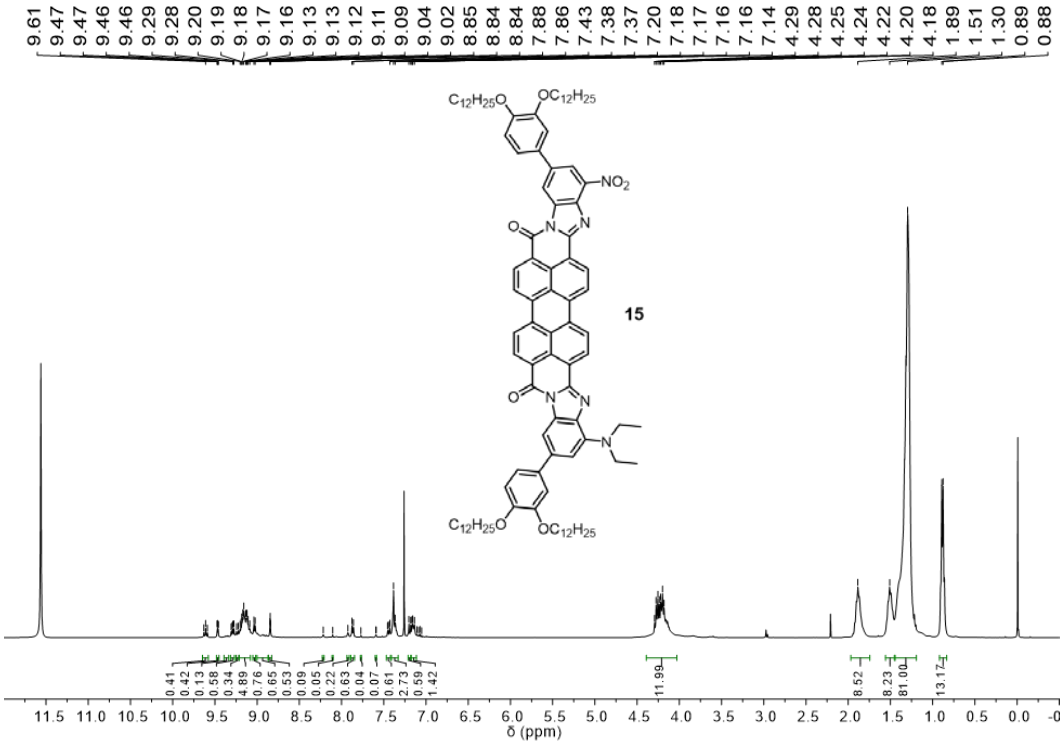

Figure S19. ^{1}H NMR of **15** (400 MHz, 10% d-TFA in $CDCl_3$, 275 K)

D: UV/vis Spectroscopy

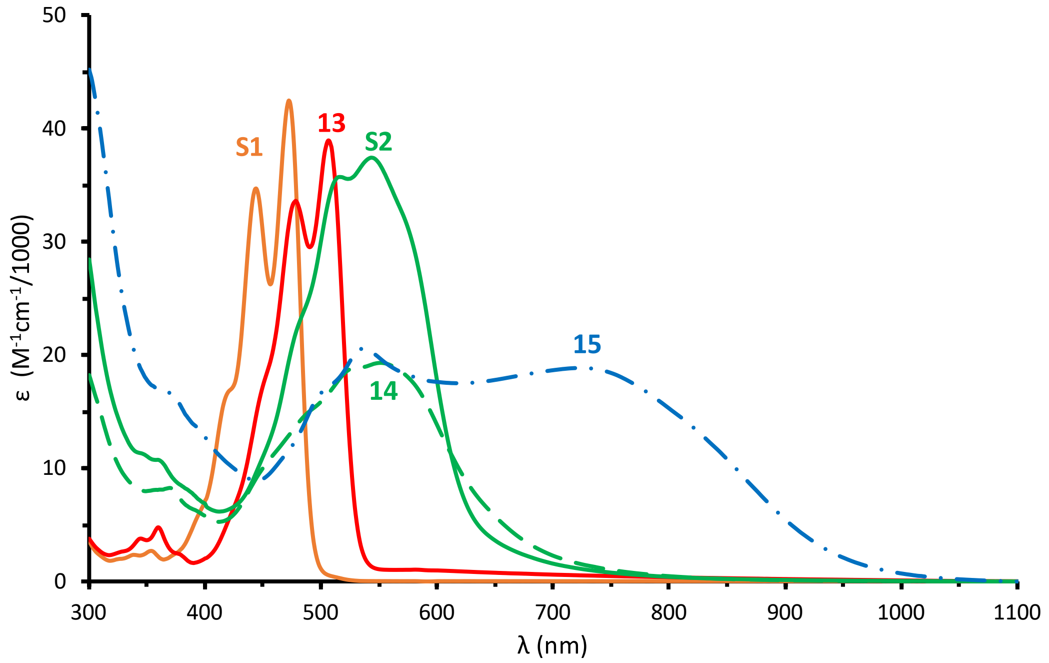

Figure S20. UV/vis of absorption of **S1** (solid orange), **13** (solid red), **S2** (solid green), **14** (dashed green), and **15** (dash-dot blue) at 50 μg/mL in $CHCl_3$.

E: DFT Calculations

Preliminary computational analysis was performed with Gaussian09 software.[5]

15 had been optimized and frequency analysis shown that the structure is confirmed to be in its minimum conformation. The UV/vis spectrum has been predicted at B3LYP/6-31G level of theory with PCM solvation model[6] and chloroform as a solvent.

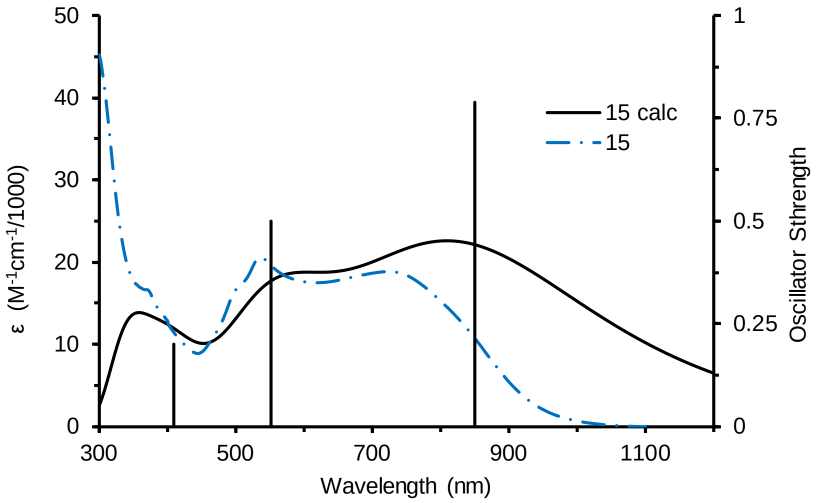

Figure S21. 15 had been optimized and frequency analysis shown that the structure is confirmed to be in its minimum conformation. The UV/vis spectrum has been predicted at B3LYP/6-31G level of theory with PCM solvation model and chloroform as a solvent. Computational spectrum (solid line) identifies three major bands at 409, 552, and 850 nm, respectively. Vertical lines are computed oscillator strength, which is a dimensionless quantity that expresses the probability of absorption. Oscillator strength and its relative strength can be compared with experimental data.

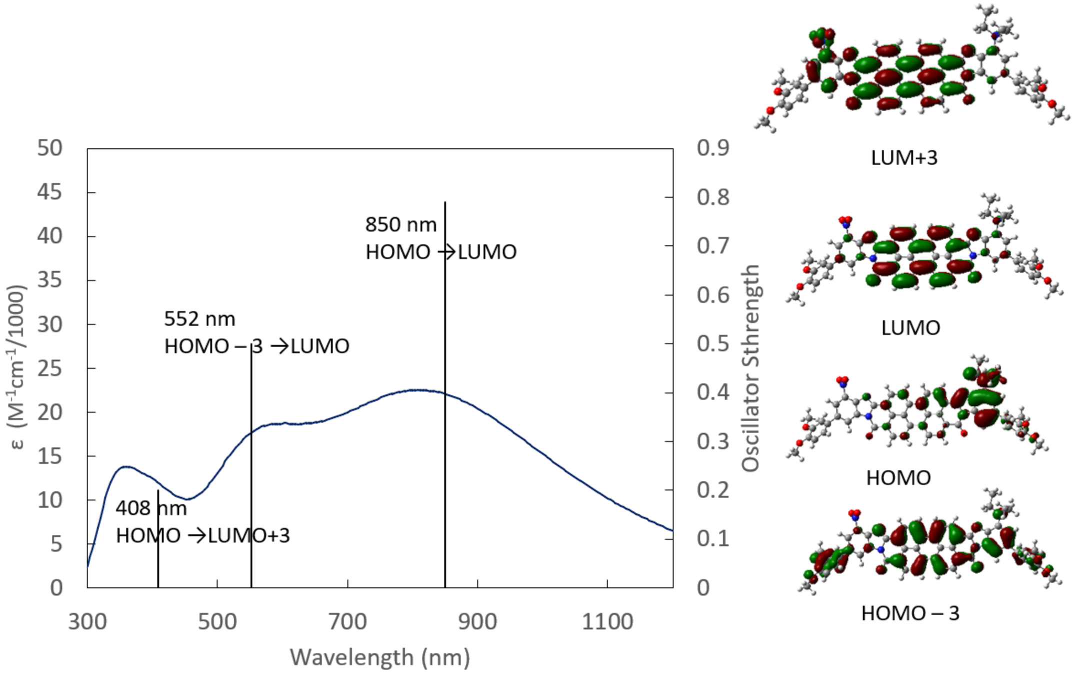

Figure S22. Predicted oscillator strength correspond to energy transitions between HOMO → LUMO + 3 (408 nm), HOMO – 3 → LUMO (552 nm), and HOMO → LUMO (850 nm).

F. Casted Films

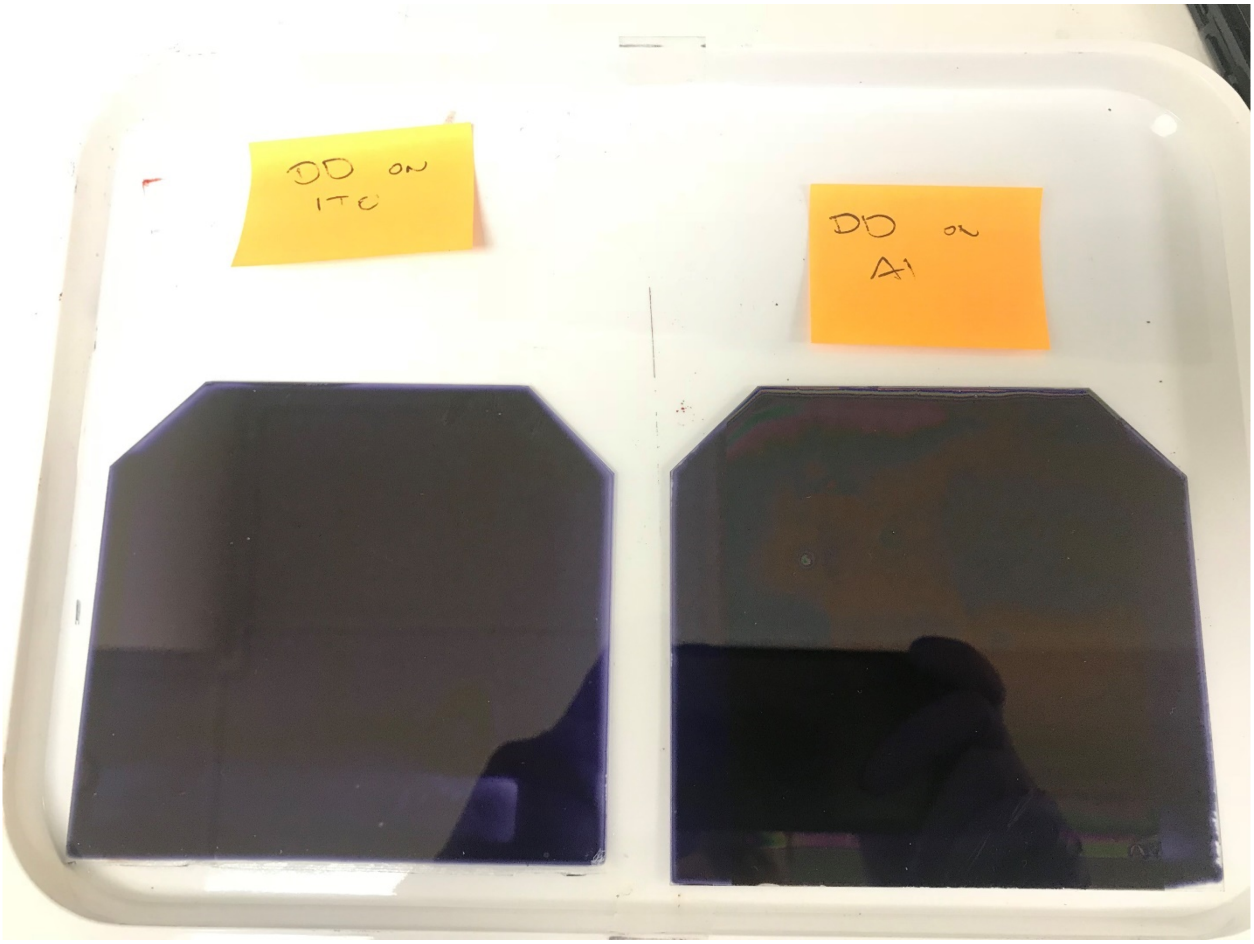

Figure S23. Films of **15** cast onto a 4 x 8-inch ITO glass slide (left) and aluminum glass slide (right) from a solution of 10 wt.% in chloroform.

E: References

1. C.W. Ong, S Liao, T.H. Chang, H. Hsu., *J. Org. Chem.*, **2004**, *69*, 3181–3185
2. B. Zhao, B. Liu, R.Q. Png, K. Zhang, K.A. Lim, J. Luo, J Shao, P.K.H. Ho, C. Chi, J. Wu, *Chem. Mater.*, **2010**, *22*, 435–449
3. X. Mo, M. Shi, J. Huang, M. Wang, H. Chen, *Dyes and Pigments*, **2008,** 76, 236-242
4. Y. Liang, H. Wang, D. Wang, H. Liu, S. Feng, *Dyes and Pigments*, **2012** 95, 260-267;
5. Gaussian 09, Revision D.02, M. J. Frisch, G. W. Trucks, H. B. Schlegel, G. E. Scuseria, M. A. Robb, J. R. Cheeseman, G. Scalmani, V. Barone, G. A. Petersson, H. Nakatsuji, X. Li, M. Caricato, A. Marenich, J. Bloino, B. G. Janesko, R. Gomperts, B. Mennucci, H. P. Hratchian, J. V. Ortiz, A. F. Izmaylov, J. L. Sonnenberg, D. Williams-Young, F. Ding, F. Lipparini, F. Egidi, J. Goings, B. Peng, A. Petrone, T. Henderson, D. Ranasinghe, V. G. Zakrzewski, J. Gao, N. Rega, G. Zheng, W. Liang, M. Hada, M. Ehara, K. Toyota, R. Fukuda, J. Hasegawa, M. Ishida, T. Nakajima, Y. Honda, O. Kitao, H. Nakai, T. Vreven, K. Throssell, J. A. Montgomery, Jr., J. E. Peralta, F. Ogliaro, M. Bearpark, J. J. Heyd, E. Brothers, K. N. Kudin, V. N. Staroverov, T. Keith, R. Kobayashi, J. Normand, K. Raghavachari, A. Rendell, J. C. Burant, S. S. Iyengar, J. Tomasi, M. Cossi, J. M. Millam, M. Klene, C. Adamo, R. Cammi, J. W. Ochterski, R. L. Martin, K. Morokuma, O. Farkas, J. B. Foresman, and D. J. Fox, Gaussian, Inc., Wallingford CT, 2016.
6. S. Miertuš, E. Scrocco, and J. Tomasi, "Electrostatic Interaction of a Solute with a Continuum. A Direct Utilization of ab initio Molecular Potentials for the Prevision of Solvent Effects," *Chem. Phys.*, **55** (1981) 117-29. DOI: 10.1016/0301-0104(81)85090-2; J. Tomasi, B. Mennucci, and R. Cammi, "Quantum mechanical continuum solvation models," *Chem. Rev.*, **105** (2005) 2999-3093. DOI: 10.1021/cr9904009; R. Improta, V. Barone, G. Scalmani, and M. J. Frisch, "A state-specific polarizable continuum model time dependent density functional method for excited state calculations in solution," *J. Chem. Phys.*, **125** (2006) 054103: 1-9. DOI: 10.1063/1.2222364
7. S. Miertuš, E. Scrocco, and J. Tomasi, "Electrostatic Interaction of a Solute with a Continuum. A Direct Utilization of ab initio Molecular Potentials for the Prevision of Solvent Effects," *Chem. Phys.*, **55** (1981) 117-29. DOI: 10.1016/0301-0104(81)85090-2; J. Tomasi, B. Mennucci, and R. Cammi, "Quantum mechanical continuum solvation models," *Chem. Rev.*, **105** (2005) 2999-3093. DOI: 10.1021/cr9904009; R. Improta, V. Barone, G. Scalmani, and M. J. Frisch, "A state-specific polarizable continuum model time dependent density functional method for excited state calculations in solution," *J. Chem. Phys.*, **125** (2006) 054103: 1-9. DOI: 10.1063/1.2222364
